# Serious Games Strategies With Cable-Driven Robots for Bimanual Rehabilitation: A Randomized Controlled Trial With Post-Stroke Patients

**DOI:** 10.3389/frobt.2022.739088

**Published:** 2022-02-17

**Authors:** Thiago Alves, Rogério Sales Gonçalves, Giuseppe Carbone

**Affiliations:** ^1^ Laboratory of Automation and Robotics, School of Mechanical Engineering, Federal University of Uberlândia, Uberlândia, Brazil; ^2^ Department of Mechanical, Energy and Management Engineering, Università della Calabria, Rende, Italy

**Keywords:** robotics, cable-driven robots, rehabilitation, bimanual, post-stroke, software, serious games

## Abstract

Cable-driven robots can be an ideal fit for performing post-stroke rehabilitation due to their specific features. For example, they have small and lightweight moving parts and a relatively large workspace. They also allow safe human-robot interactions and can be easily adapted to different patients and training protocols. However, the existing cable-driven robots are mostly unilateral devices that can allow only the rehabilitation of the most affected limb. This leaves unaddressed the rehabilitation of bimanual activities, which are predominant within the common Activities of Daily Living (ADL). Serious games can be integrated with cable-driven robots to further enhance their features by providing an interactive experience and by generating a high level of engagement in patients, while they can turn monotonous and repetitive therapy exercises into entertainment tasks. Additionally, serious game interfaces can collect detailed quantitative treatment information such as exercise time, velocities, and force, which can be very useful to monitor a patient’s progress and adjust the treatment protocols. Given the above-mentioned strong advantages of both cable driven robots, bimanual rehabilitation and serious games, this paper proposes and discusses a combination of them, in particular, for performing bilateral/bimanual rehabilitation tasks. The main design characteristics are analyzed for implementing the design of both the hardware and software components. The hardware design consists of a specifically developed cable-driven robot. The software design consists of a specifically developed serious game for performing bimanual rehabilitation exercises. The developed software also includes BiEval. This specific software allows to quantitatively measure and assess the rehabilitation therapy effects. An experimental validation is reported with 15 healthy subjects and a RCT (Randomized Controlled Trial) has been performed with 10 post-stroke patients at the Physiotherapy’s Clinic of the Federal University of Uberlândia (Minas Gerais, Brazil). The RCT results demonstrate the engineering feasibility and effectiveness of the proposed cable-driven robot in combination with the proposed BiEval software as a valuable tool to augment the conventional physiotherapy protocols and for providing reliable measurements of the patient’s rehabilitation performance and progress. The clinical trial was approved by the Research Ethics Committee of the UFU (Brazil) under the CAAE N° 00914818.5.0000.5152 on plataformabrasil@saude.gov.br.

## 1 Introduction

Stroke is the leading cause of disability. It leaves a significant number of individuals with motor and cognitive deficits (World Health Organization, 2021). The paralysis of the upper limb is the most frequent consequence of brain injury. Rehabilitation training is the most effective way to reduce motor impairments in stroke patients. However, conventional rehabilitation sessions can be shorter than ideal also due to shortage and costs of therapists. Moreover, rehabilitation sessions can be strongly affected by therapist skills as well as they are not providing objective measurement of the patient’s performance and progress (Hatem et al., 2016). It is to note that the recovery of the impaired limb is usually not the ultimate therapy goal. Instead, the key goal should be to recover the ability to perform activities of daily living (ADL) which are predominantly bimanual tasks. The bimanual ADL movements involve a simultaneous coordinated use of both hands, and they are the most difficult tasks to be performed by stroke patients (Eggers, 1984; Cardoso et al., 2017).

Different types of robots are being studied for rehabilitation tasks. However, conventional robots usually are expensive, have rigid structures, and are complex to operate. They have several safety issues when applying them to rehabilitation (Mao, 2015). An interesting low-cost alternative approach can be based on cable-driven robots. Cable-driven robots consist of a mobile platform that is linked to a fixed platform though flexible cables. The cables lengths are variable (can be extended or retracted) to control position and orientation of a mobile platform, while preventing cables to became slack. Cable-driven robots have several advantages such as small and lightweight moving parts, a relatively large workspace. They also have intrinsically safe features due to the low inertia and flexibility of cables. This is allowing safe manipulation in close human-robot interactions. The relatively large workspace of these devices make them suitable and adaptable to different patients and training protocols. Additionally, cable-driven robots are cheap, have simple maintenance and lightweight structures that can be easily reconfigured. In the clinical aspect, the use of cables instead of rigid links makes the patient feel less constrained. This is resulting in an improved user acceptance (Gonçalves et al., 2020). All those characteristics make cable-driven robots as ideal candidates for rehabilitation and even for self-treatment at home, as it is proposed for example in Nijenhuis et al. (2015). However, the existing cable-driven robots are mostly unilateral devices allowing the rehabilitation only of the most affected limb. This leaves unaddressed the rehabilitation of bimanual activities, which are predominant within the common ADL (Mehrholz, 2012; Cardoso et al., 2017).

Effective rehabilitation after stroke should be early, intensive, and repetitive. The latter can lead to problems with the patient engagement, since conventional rehabilitation exercises is often monotonous and boring. A new paradigm is emerging in the field of rehabilitation, characterized by the systematic use of computer games, called serious games. Serious games aim to provide an interactive experience and generate a high level of engagement in patients during the rehabilitation. Serious games motivate the patient to perform a prescribed sequence of rehabilitation exercises in a more attractive and engaging way, allowing to soften the feeling of tiredness during the exercises (Appel et al., 2014; Moretti et al., 2014). In this way, robotic therapy can be combined with serious games and it allows an intensive, encouraging, and motivating environment for patients (Proença et al., 2018; Aguilar-Lazcano et al., 2019). Additionally, it can improve and adjust the therapy progress by recording patient activity information such as exercise durations, velocities, and forces. Quantitative performance data can be a very valuable tool for providing an objective measure of the progress and outcomes of therapy, as proposed in (Ceccarelli and Romdhane, 2010; Hussain et al., 2017; Hocoma, 2019; InMotion, 2021). The above mentioned advantages give a strong motivation for this work where it is proposed, analyzed, and experimentally validated a combination of a cable-driven robot with a serious game interface.

This paper is organized as follows: Section 2 gives a literature review and definition of design requirements focused on both hardware and software aspects; then, Section 3 outlines the design process and main mathematical model of the proposed robot in combination with a specifically developed serious game for bimanual rehabilitation exercises. Section 4 outlines the main features of the proposed BiEval software to evaluate patients’ performance and to monitor their progress based on quantifiable parameters. Section 5 presents a pilot Randomized Controlled Trial (RCT) to test the effectiveness of BiCAR and to quantitatively assess the training and rehabilitation stage.

## 2 Design Requirements and Literature Review

This section gives insight on the current state of art aiming at identifying the main design requirements towards a novel robotic solution both from hardware and software viewpoints.

### 2.1 Cable-Driven Robots for Rehabilitation

In the last decade several cable-driven robots have been proposed for rehabilitation tasks. Some significant common features are low-inertial forces in its links while the cables can break at a designed tension force. Additionally, the requirements for portability, safety, comfort, and ease of manipulation, can be met by these devices as highlighted for example by Ceccarelli and Romdhane (2010), Carbone and Ceccarelli (2016). The NeReBot (NEuro REhabilitation roBOT) (Rosati et al., 2005; Stefano et al., 2014) is a 3 degrees of freedom (DOF) robot. It focuses on the treatment of the stroke acute phase. In this device, electric motors actuate the cables that support the patient’s limbs. MariBot (Marisa robot) (Rosati et al., 2005; Rosati et al., 2017) is an evolution of NeReBot with 5 DOF. It uses a similar principle of NeReBot, but it has 2 additional DOFs (SCARA robot architecture). The Lawex is a cable-driven robot solution with four cables that allows three DOFs for upper limb rehabilitation. This device has four DC motors, each one is equipped with an encoder that allows speed and direction control performed by a microcontroller. The difference between the desired and actual position defines the motor speed input. A user interface defines the motion trajectories by setting up a list of point coordinates that one wishes to reach versus time (Carbone et al., 2017; Laribi and Carbone, 2019; Boschetti et al., 2019). The CUBE is a 6 DOFs cable-driven manipulator for rehabilitation with 6 cables/motors. This device focuses on both upper and lower limbs and the user wears a ring (mobile platform) as a wristband. It is controlled in the same way as mentioned for Lawex (Cafolla et al., 2019). The CADEL is a cable-driven elbow rehabilitation device with a focus on a low-cost prototype design and its design evolution (L-CADEL v2) also considers the patient’s comfort and ease of use (Ceccarelli et al., 2021). The CABLEankle is a cable-driven wearable device for motion ankle exercising in rehabilitation training (Russo and Ceccarelli, 2020). The DIEGO^®^ developed by Tyromotion, Diego, (2021) is a cable-based device for unilateral and bilateral rehabilitation of shoulder and arm. DIEGO^®^ is adaptable to the need of the patients and uses a gravity compensation control that assist them as necessary. DIEGO^®^ also has games that stimulates and facilitates training.

### 2.2 Bimanual Devices and Rehabilitation Strategies

The Driver’s Simulation Environment for Arm Therapy (SEAT) is a device with only 1 DOF, a servomotor and a steering wheel. Each arm has its force measured independently. In a specific mode of operation, both upper limbs are required to move. If he or she tries to use only the less impaired upper limb, a warning (steering wheel stiffness proportional to less impaired arm use) reminds him/her not to do so. The steering wheel stiffness is proportional to the less impaired arm use, which leads to an immediate awareness of strong-arm use and corrective action by stroke patients (Johnson et al., 2005). An Adaptive Bimanual Robotic Training for driving tasks was developed in Trlep et al. (2011). This system uses the HapticMASTER robotic system, with 3 DOFs plus an active joint. The handlebar that is mounted on the robot end-effector rotates like a steering wheel and measures the force given by each arm in an independent way. A flight simulator game is used with a gravity compensation mechanism but during the training the patients are stimulated to also use the weaker limb. The System for Independent Task-oriented Assessment and Rehabilitation (SITAR) uses real objects instead of interacting with a virtual environment. Thus, patients interact with smart-instrumented objects and one can perform kinetic and dynamic measurements to assess user progress and to integrate games that simulate daily tasks (Aguiar et al., 2017; Cardoso et al., 2017). Among the developed games, the PizzaGame aims to emulate the task of assembling a pizza. In one of the stages, it is necessary to open the dough with the aid of a bimanual module that is similar to a kitchen roll. But, instead of rolling, the object would slide over a table, simplifying the acquisition of the involved forces (Aguiar et al., 2017). Cardoso et al. (2017) shows a robotic version of a bicycle handlebar that has been developed in the SITAR context. This device has sensors to measure hand grip forces, speed and range of motion when turning the handlebar. An electric motor applies torque resistance when the handlebars are moved. It is hypothesized that therapies which involve cooperation among limbs, like cycling, may result also in recovery of others distinct activities like walking and unilateral manipulation, besides improving the general patient health.

### 2.3 Neurostimulation on Stroke Rehabilitation

There is growing hope to discover a safe/effective way to induce the process of neuronal growth and repair injured brain areas (Valentin, 2017). The skills of the brain could be improved in several ways: through electrostimulation on the affected area with transcranial direct-current stimulation (tDCS) or any other neurostimulation task that improves the cognitive functions. Serious games can be one of the best ways for brain rehabilitation (Ballesteros et al., 2015; Dedoncker et al., 2016). Studies suggest that a combination of rehabilitation methods can help with personalized treatment strategies, particularly when using some type of non-invasive brain stimulation such as transcranial stimulation or serious games (Auriat et al., 2015).

Repetitive Transcranial Magnetic Stimulation (rTMS) can be applied to increase the cortical excitability before an intervention as skill motor practice (Brodie et al., 2014). The rTMS can significantly improve the dexterity of the affected hand in stroke patients (Lüdemann-Podubecka et al., 2015), however, it is important to note the countless variables present in these studies such as the lesion’s size and location, stroke chronicity and the dominant hemisphere, which requires further research with new technologies and combined techniques to fully understand their effects on stroke recovery (Auriat et al., 2015).

### 2.4 Features and Serious Games Requirement for Stroke Rehabilitation

This subsection presents an outlook of the key design characteristics and recommendations for the development of serious games for stroke rehabilitation. Games for therapeutic purposes must be simple but challenging enough to keep patients engaged without developing any type of frustration or boredom (Ferreira and Menezes, 2020). Stroke patients must be provided with simple gameplay and the ability to recover quickly from any failure in the game, with little to no mention of gaming concepts such as lose, die, or fail. Rather the progress should be represented in the form of advancing levels and encouraging positive interactions. By handling failure in a positive way, patients are more likely to remain engaged and not feel that failure in the game stems from their impaired physical abilities (Burke et al., 2009). Contextualizing the game play is imperative, so that any player actions have meaning and relevance. Customized profiling, while difficult to implement, can eventually improve rehabilitation outcomes. Further, due to the disparity in cognitive and motor characteristics of each patient, adaptation and customization is essential. Delivering feedback across a number of modalities (sound, visual or haptic) will enhance the engagement and the experience of the stroke patient (Mubin et al., 2020).

Subjects that play action games improve the following abilities: focus, motor dexterity, and solving mental problems (Mishra et al., 2016). The target-based games and Tetris-like games are considered one of the most successful type of games and are adequate for the intended purpose of rehabilitation. These type of games do not only allow the execution of movements that strengthen patients’ muscles of the arms and shoulders, but also produce the necessary stimulation of their cognitive activity and sensory inputs (Ferreira and Menezes, 2020; Mubin et al., 2020). Several metrics were proposed in the literature to assess the patient’s performance during the playing. The main metrics used in the studies are medical scale evaluation, range-of-motion comparison, game score, and physiotherapist evaluation (Aguilar-Lazcano et al., 2019). The score can be used to quantitatively evaluate the patient’s evolution, which is an information of great value for physical therapists. The score is useful also for the game itself to calibrate attributes (interface aspects, game difficulty, assistance) in order to equalize and adapt the conditions for any patient and disability (Caurin et al., 2011; Appel et al., 2014; Moretti et al., 2014).

### 2.5 Evaluation Software in Rehabilitation

Stroke patients frequently have their motor functions evaluated by tasks of drawing/tracing circles (Dipietro et al., 2007; Hussain et al., 2017; InMotion, 2021). The shoulder and elbow abnormal coupling of stroke patients influences circle drawing performance, as they produce ellipses shapes instead. The training generates an increased hemiparetic arm work area and circle drawing performance (Krabben et al., 2012), even when subjects do not train for circle drawing (Krebs et al., 2009). Even just point-to-point movements, demonstrate skill learning in joint movements coordination.

The InMotion ARM™ is a robotic arm with two active DOFs. It is the most researched device for upper limb rehabilitation with evidence of improvements in the FIM (Functional Independence Measure). This device uses circle and point to point evaluation tests included in the software InMotion Eval™ to measure the range of motor coordination, joint independence, and coordinated movement planning. Stroke patients’ evaluation at admission and discharge following robotic therapy with this device shows improvements in a drawn circle pattern task. Quantifiable measures are obtained by the InMotion Eval™ such as shoulder stabilization, smoothness of movement, mean and maximum speed allows clinicians to distinguish true recovery from compensation. The patient progress is also correlated with traditional assessment scales such as Fugl-Meyer. This device, among others, has sets of therapeutic exercises with serious games for motor planning, coordination, and attention (InMotion, 2021). The ArmeoBoom^®^ is providing support for the arms. It is designed for domestic use allowing exercises in a three-dimensional workspace. It has serious games which use a webcam and a potentiometer. Whenever there is an improvement in elbow extension, the support level is reduced. Feedback and performance evaluation tools are available through Armeo^®^ software that provides the patient’s progress (Hocoma, 2019).

### 2.6 Summary Comparison of Rehabilitation Devices

The main characteristics of the devices presented in this section review are summarized in Table 1. It can be seen from this table that most devices for rehabilitation purposes are serial robots or complex cable-actuated devices. The serial robot devices are heavy, expensive, and inaccessible to the general population as they require trained operator, specialized clinics and are sold for thousands of dollars. The reported cable-actuated devices are expensive devices that use multiple motors and cables, plus a complicated control. The high cost and complexity decrease the presence of such devices in rehabilitation clinics especially for poor or underdeveloped countries.

**TABLE 1:** Summary of the main characteristics of the devices presented in the review.

Device	DOFs/Arm motions	Joints involved	Type	Control strategy	Actuator/Mechanism	Cables number
*InMotion®*	2 DOF	Shoulder and Elbow	Planar Robot/Vertical Gravity Compensation	Assistive/Resistive	DC Motors	-
*ArmeoBoom*	3D Workspace	Shoulder and Elbow	Gravity Compensation	Passive/Active	Sling system	2
*NeReBot*	3 DOF	Shoulder and Forearm	Cable-Driven Robot	Passive	3 DC Motor	3
*MariBot*	5 DOF	Shoulder and Forearm	Cable-Driven Robot	Passive	3 gear +2 joint motors	3
*DIEGO*	3D Workspace	Arm-Shoulder	Gravity Compensation Cable-Driven Robot	Bilateral/Assistive	2 Motors each side	2 each side
*Lawex*	3 DOF (trigonal prism workspace)	Shoulder and Elbow	Cable-Driven Robot	Assistive	DC Motor	4
*CUBE*	5 DOF	Upper/Lower Limbs	Cable-Driven Robot	Assistive	6 DC Motor	6
*SEAT*	1 DOF	Shoulder and Elbow	Bimanual Robot	Bimanual/Resistive	not specified	-
*ABRT*	3DOF +1 active joint	Shoulder and Elbow	Bimanual Robot	Bimanual/Resistive	not specified	-

Additionally, most of these cable driven robots are unilateral devices that can provide treatments focusing on the most affected limb while neglecting bimanual activities, which are predominantly related to ADL. Even though sometimes bimanual training does not yield a superior primary outcome it has benefits such as increased daily use of the paretic side and recovery from other activities (Eggers, 1984; Malabet et al., 2010; Cardoso et al., 2017). Therefore, this paper presents a low-cost cable-driven robot to be applied in the bimanual rehabilitation, that is, for therapies which require simultaneous use of both hands in a coupled way. This device is expected to transfer the training skills to ADL and teach patients to use both arms more evenly.

## 3 Design of a Novel Bimanual Cable-Actuated Robot

The following subsections report the design of the proposed novel robot from hardware/software and mathematical modelling viewpoints.

### 3.1 Hardware Design and Control for the Cable-Actuated Robot

**FIGURE 1:**
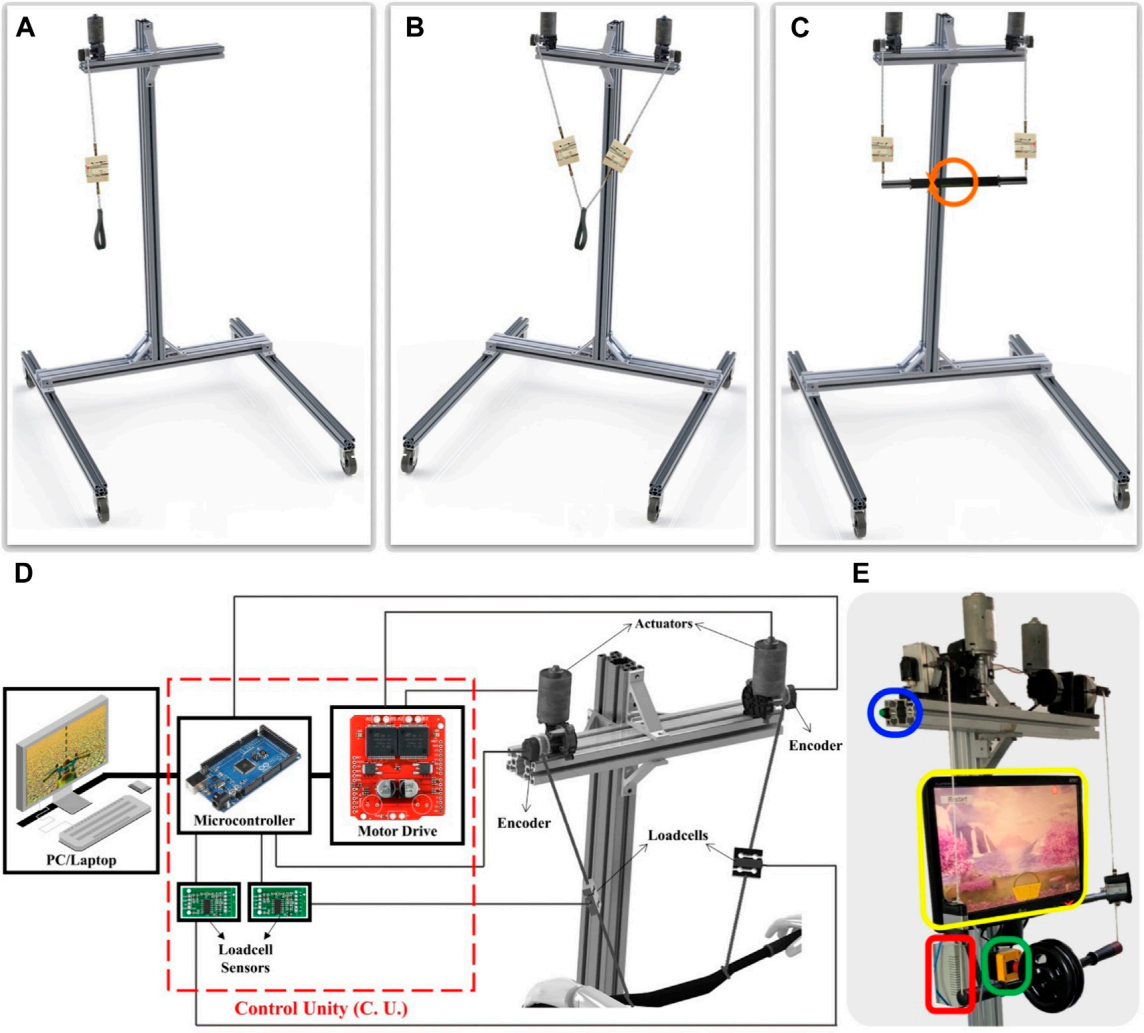
CAR: **(A)** 1 DOF unilateral, **(B)** 2 DOF unilateral and **(C)** 2 DOF Bimanual setups, **(D)** connections schematic, **(E)** components highlight: control unit (red), emergency (green) and action (blue) buttons, feedback unit (yellow).

The Cable-Actuated Robot (CAR), Figure 1, was developed at the Laboratory of Automation and Robotics of the Federal University of Uberlandia, Brazil. It is a cable-driven structure with 2 DOFs. Depending on the setup (Figures 1A–C) the end-effector is a handle/strap or a handlebar. The structure consists of an aluminum fixed frame, DC motors, load cells, and rotary encoders. The motors are powered by a 24 V DC power supply, the nominal torque and power are respectively 10 N m (48 N m peak) and 46 W; rotational speed is up to 45 RPM. Each load cell has a 20 kgf capacity and the resolution of the measuring system is 0.025 kgf. The encoders are incremental and produce 500 ppr (pulses per revolution). To amplify and condition the load cell signal, two HX711 converter modules (one per load cell) have been added with 24-bit resolution and 80 Hz refresh rate. The connection layout of these components is shown in Figure 1D. An overview of the CAR main components is shown in Figure 1E: the control unit, shown in Figure 1D, was miniaturized and mounted in a box as shown in red; for safety reasons, an emergency button has been included to cut off the power supply and stop all motors when pressed (green); an action button quickly starts/stops an action/game (blue); finally, a feedback unit (monitor) is attached to the T-frame to improve the user’s posture in front of the device (yellow). This is avoiding possible injuries due to misalignment of the chair/user and monitor. In this setup this device has 2 DOFs and it is used bilaterally with a coupling between the two sides of the human body. This configuration will be used in coupled upper limb rehabilitation for bimanual therapy. The control is performed by a microcontroller (Arduino MEGA), MATLAB software and a VNH2SP30 motor driver (2 actuators/30 A). The microcontroller sends the sense of rotation and the speed using PWM modulation to reach the desired position. Additionally, the load cells can be used to measure the patient engagement and to analyze his/her intention of movement direction.

This device has different control strategies for allowing the implementation of several rehabilitation strategies. Fully passive rehabilitation exercises are performed when the movement is imposed externally to the patient for increasing joints amplitude, this device applies a position proportional control. The actuators speed input depends on the current and the desired position difference and the speed decreases while moving until the desired position is reached. Active training exercises are achieved when a movement is performed voluntarily. The active training is more beneficial for the functional recovery (Lotze et Al., 2003). To prevent hindering the patient’s recovery due to a lesser participation (Zhang et al., 2013), the assistance is applied only when and as much needed (assist-as-needed strategy) based on the patient’s performance and progress. Movement can even be hampered according to this progress (active-resistive strategy). Rehabilitation movements can also be classified as unilateral, when using only the affected limb (paretic side) or bilateral, when using both sides. The bimanual movement is a specific bilateral movement, in which both hands are used simultaneously in a coupled way (Malabet et al., 2010). To allow all the above-mentioned rehabilitation movements this device has 3 main possible setups:

• I - Unilateral with 1 DOF: in this setup, Figure 1A, the device has 1 DOF and is used unilaterally. Movement performed by this device setup is only translation over *y*-axis.
• II - Unilateral with 2 DOFs: in this setup, Figure 1B, the device has 2 DOFs, but also is used unilaterally. There is no coupling between the human body sides. Movements performed by this device setup are translation over *x*-axis and translation over *y*-axis. These DOFs combined allow for planar trajectories.
• III - Bimanual with 2 DOFs (BiCAR): in this setup, Figure 1C, the device has 2 DOFs and is used bilaterally, however there is coupling between the two sides of the human body. This configuration will be used in coupled upper limb rehabilitation, the bimanual therapy.

**FIGURE 2:**
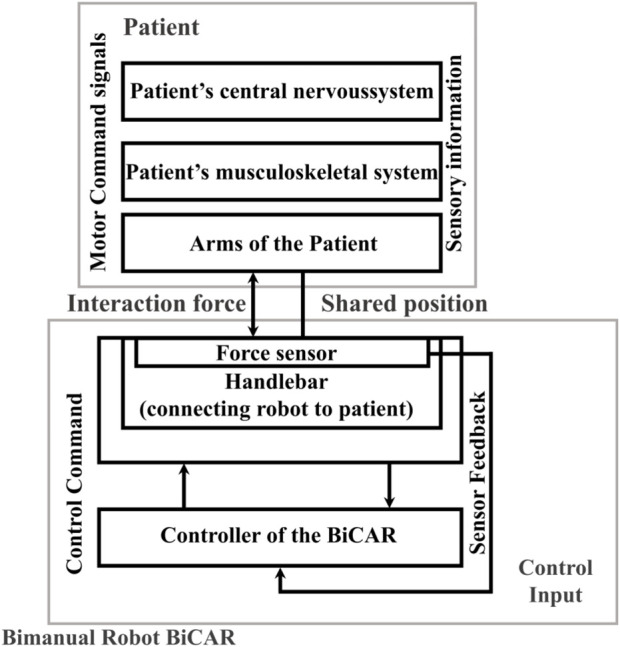
System drawing of the bimanual robot for rehabilitation BiCAR that applies a patient-cooperative shared control strategy. Adapted from standard IEC 80601-2-78:2019 (IEC, IEC, 2019).

From the standard IEC 80601-2-78:2019 (IEC, IEC, 2019), the rehabilitation robot for arms movements may use a patient cooperative strategy which assists him/her only when necessary to maximize his efforts. Figure 2 shows a schematic of the BiCAR with the assist-as-needed approach, which applies a shared control with the patient. The patient and BiCAR controllers interact each other and feedback is measured by means of the interaction force by each arm with the handlebar.

For the bimanual games, which will be shown in Section 3.3, the control is based on a force map in which the patient rotates the handlebar by applying a different force on the right and left side as equal to ΔF. Assistance is applied when ΔF decreases. The assistance uses a performance-based strategy, i.e., it is activated/increased if the patient underperforms and deactivates/decreases when patient performance improves. Thus, the device only assists the patient when and as needed (assist-as-needed), automatically adjusting game difficulty according to the performance, and returning the control to patient as soon as a progress is achieved. The bimanual serious games are expected to encourage post-stroke patients to improve symmetrical use of both arms and consequently improve their recovery in ADLs.

### 3.2 Mathematical Model

For the first setup of the CAR there is no need for modeling as the only movement performed is the translation over *y*-axis, thus, the cable length *L* must be calculated to reach the desired position of the mobile platform P ( $x_{p},y_{p},z_{p}$ ). As $x_{p}$ and $z_{p}$ is constant:

$$\begin{array}{c} L=y_{p} \end{array}$$ (1)

**FIGURE 3:**
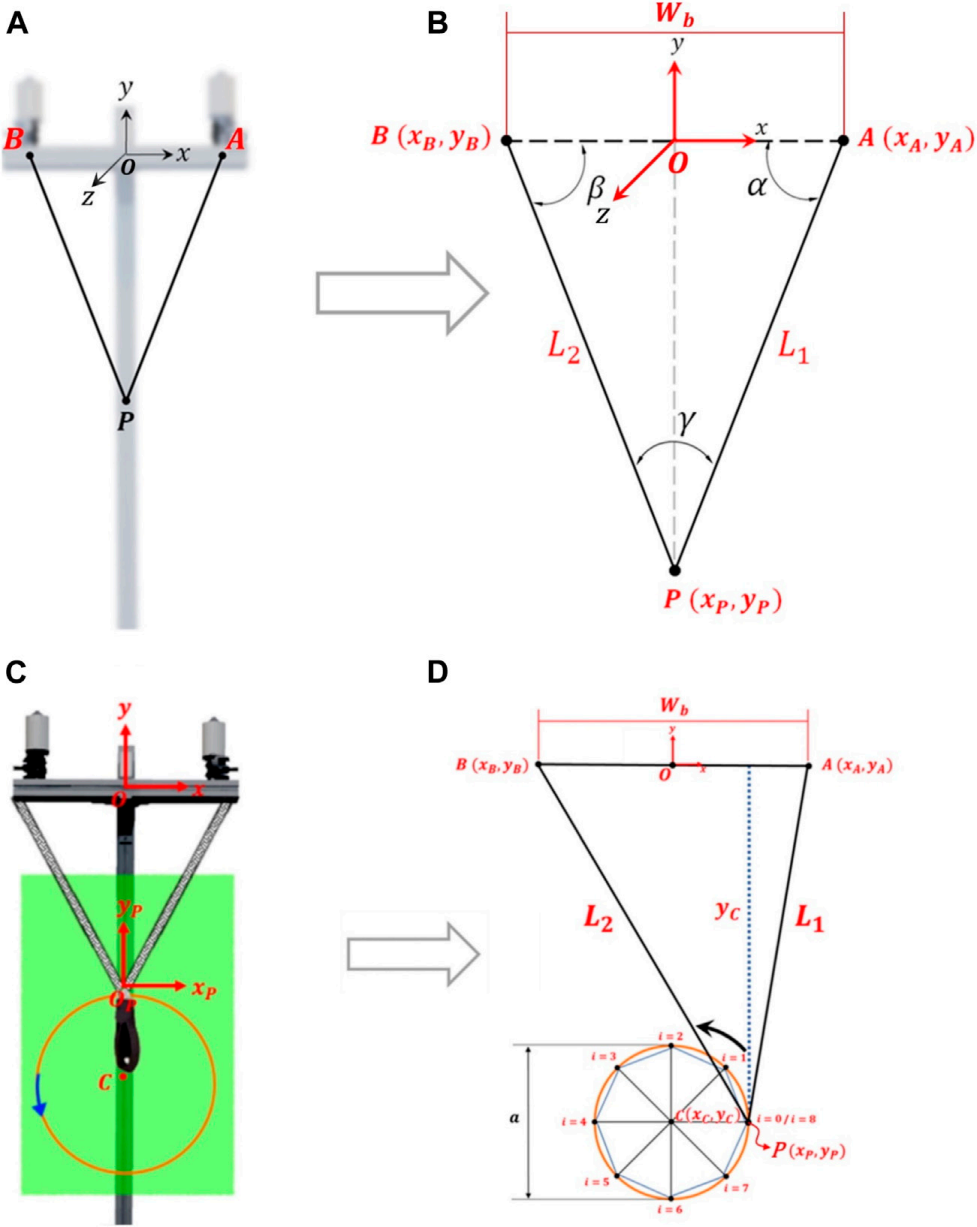
Trajectories: **(A)** computational model and **(B)** schematic to obtain the mathematical model for planar trajectory, **(C)** and **(D)** for circular trajectory (example with 8 points).

For planar trajectories (setup II), since the cables must always pull, they are modeled as rigid bodies. Accordingly, two cables will form a triangle with the fixed and the mobile platform as shown in the scheme of Figure 3. In the mathematical model, the lengths of the cables are represented by $L_{1}$ and $L_{2}$ , **A** and **B** are the points where the cable $L_{1}$ and $L_{2}$ are attached to the fixed platform respectively and P is the desired position on the mobile platform; the distance between **A** and **B** is given by $W_{b}$ . Again, $z_{p}$ is constant for planar trajectories. So, from a desired position **P** ( $x_{p},y_{p}$ ), the controller drives the motors so that the cables reach the positions with length $L_{1}$ and $L_{2}$ , respectively:

$$\begin{array}{c} L_{1,2}=\frac{y_{p}}{sin\;\left(tan^{-1}\left(\frac{y_{p}}{x_{p}\pm \frac{W_{b}}{2}}\right)\right)} \end{array}$$ (2)

The circular trajectory, Figure 3C, is a particular case of the planar trajectory. Therefore, its modeling assumes the same considerations. However, a specific approach facilitates this modeling. A circular trajectory can be defined with a movement amplitude (diameter *a*) and the center of the trajectory in **C** ( $x_{c},\;y_{c}$ ), Figure 3D. The desired position **P** ( $x_{p},\;y_{p}$ ) is given by Eq. 3 and Eq. 4, respectively:

$$\begin{array}{c} x_{p}\;=\;\;\;x_{c}\;+\;\frac{a}{2}cos\;\theta \end{array}$$ (3)

$$\begin{array}{c} y_{p}\;=\;\;\;y_{c}\;+\;\frac{a}{2}sin\;\theta \; \end{array}$$ (4)

where θ is the angle within the circular trajectory and can be discretized as:

$$\begin{array}{c} \theta \;=\;i\;\times \;\frac{2\pi }{N},for\;\;i\;=\;1,2,3,\ldots ,N \end{array}$$ (5)

where *N* is the number of discretization points and *i* is the index. A spline can be used to interpolate the points and produce smoother movements. Thus, cable lengths are calculated using Eq. 6:

$$\begin{array}{c} L_{1,2\;}=\frac{y_{C\;}+\;\frac{a}{2}\;sin\;\left(i\;\frac{2\pi }{N}\right)}{sin\;\left(tan^{-1}\left(\frac{y_{C}+\frac{a}{2}sin\left(i\;\frac{2\pi }{N}\right)}{x_{C}+\frac{a}{2}cos\left(i\;\frac{2\pi }{N}\right)\pm \frac{W_{b}}{2}}\right)\right)} \end{array}$$ (6)

For polygonal trajectories such as triangles, quadrilaterals, and pentagons, it is enough to decrease the number of discretization points to 3, 4 and 5 respectively. If necessary, a rotational angle α can be added in Eq. 5 to rotate the polygon; this is shown in Eq. 7.

$$\begin{array}{c} \theta \;=\;i\;\times \;\frac{2\pi }{N}+\alpha ,for\;\;i\;=\;1,2,3,\ldots ,N \end{array}$$ (7)

**FIGURE 4:**
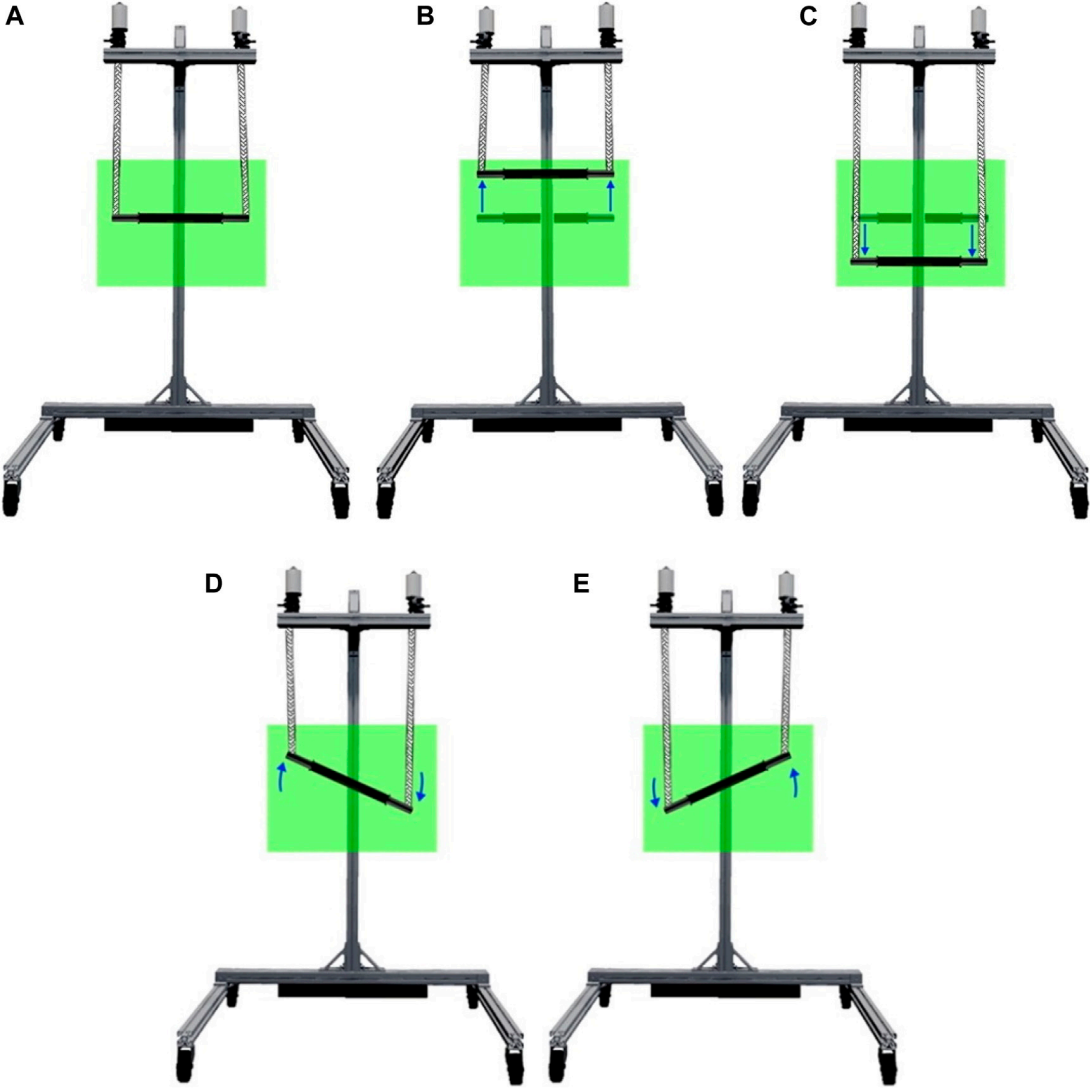
Bimanual robot DOF, **(A)** initial position, **(B)** upward movement, **(C)** downward movement, **(D)** clockwise movement, **(E)** counterclockwise movement.

Finally, in bimanual setup the movements performed by the BiCAR are translation over *y*-axis and rotation over *z*-axis as shown in Figures 4B,C and d-e, respectively. To perform these movements, were used two actuated cables connected to the same end-effector, which can be a handlebar or a steering bar, set up to allow planar trajectories. In this way there is coupling between the cables, and also related to both human body sides. The dynamics and inertial effects can be neglected due to negligible moving mass and low speed movement. Figures 4A–C shows an example of vertical movement. If the cable lengths decrease of the same amount (both motors roll the cables equally) the bar moves up, Figure 4B. Otherwise, if they increase of the same amount (unroll equally) the bar descends, Figure 4C. This device is under-constrained and therefore the weight/gravity is required for achieving the downward movement. In this case, there is an additional weight concentrated in the middle of the handlebar, Figure 1E, which is used to maintain positive cable tension and favor downward movement. For rotational movements are necessary to decrease the length of one cable while increasing the length of the other. Figures 4D,E shows examples of rotation movements, decreasing the left cable length and increasing the right one the bar rotates clockwise (CW), Figure 4D. Similarly, decreasing the right and increasing the left one the bar rotates counterclockwise (CCW), Figure 4E.

**FIGURE 5:**
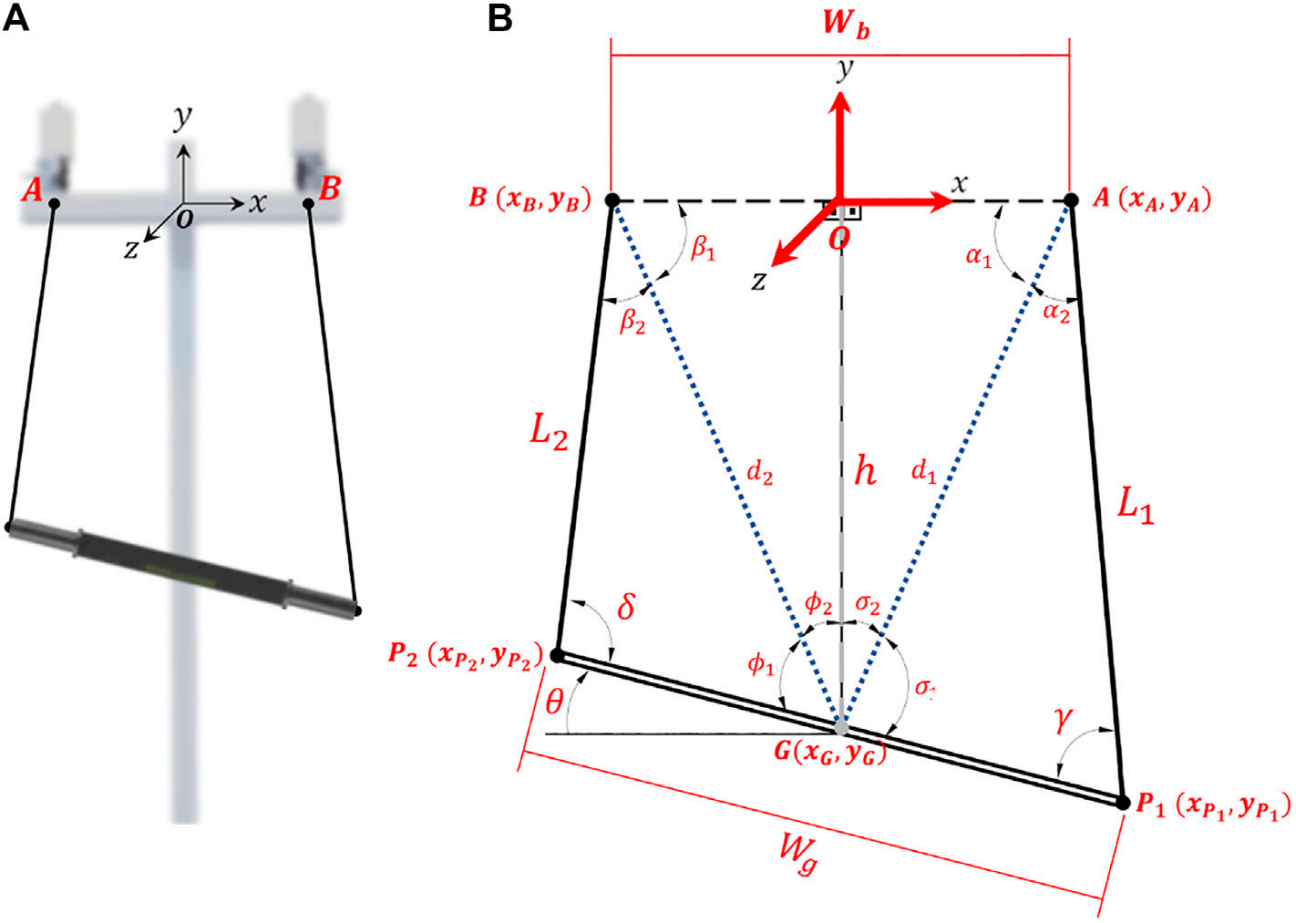
Bimanual robot device: **(A)** computational model; **(B)** schematic to obtain the mathematical model.

For modeling, Figure 5 the desired bar height/orientation (h/ θ ) are considered as input values, $W_{b}$ (distance between module) is considered centrally aligned with the $O_{XYZ}$ reference; $W_{g}$ (handlebar width) and $W_{b}$ are considered horizontally aligned also. Thus, both cables lengths, $L_{1}$ and $L_{2}$ , are given by Eq. 8:

$$\begin{array}{c} L_{1,2}=\sqrt{{\left(W_{b}/2\right)}^{2}+h^{2}+W_{g}\left(W_{g}/2-\left(\sqrt{{\left(W_{b}/2\right)}^{2}+h^{2}}\right)cos\left(\text{π}/2\pm \text{θ}-tan^{-1}\left(W_{b}/2h\right)\right)\right)} \end{array}$$ (8)

For the serious games that will be shown in Section 3.3 the inverse modeling is necessary. The bar orientation ( θ ) is given as cables lengths function as shown in Eq. 9. The cable lengths are obtained by the encoders, considering the system approximately linear, despite the cables unrolling.

$$\begin{array}{c} \Theta =cos^{-1}\left(\frac{-4L_{1}^{2}+W_{b}^{2}+2W_{g}^{2}+4h^{2}}{4W_{g}\sqrt{W_{b}^{2}/4+h^{2}}}\right)-cos^{-1}\left(\frac{-4L_{2}^{2}+W_{b}^{2}+2W_{g}^{2}+4h^{2}}{4W_{g}\sqrt{W_{b}^{2}/4+h^{2}}}\right) \end{array}$$ (9)

### 3.3 Serious Games for Bimanual Rehabilitation

Several serious games were developed for the CAR device for its different setups and will be shown in this subsection.

**FIGURE 6:**
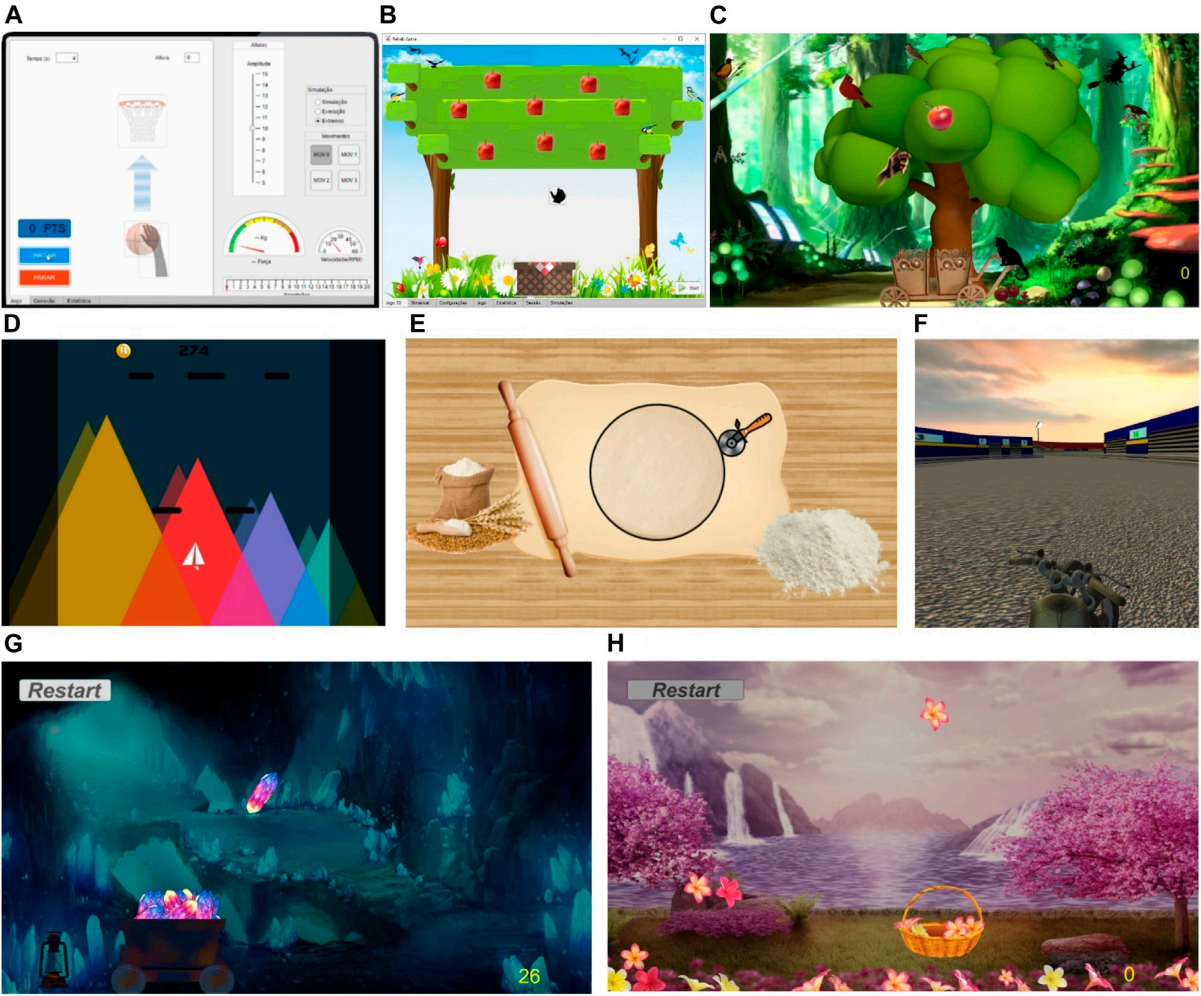
Main screen of the serious games: **(A)** Rehab Basketball, **(B)** Square Apple, **(C)** Grabbing Apple, **(D)** Paper Plane (escape mode), **(E)** Round Pizza, **(F)** Motorcycle, **(G)** MineCart, and **(H)** Sakura Flowers.

The Rehab Basketball, Figure 6A, was designed for the CAR in setup I. This game is very simple and was completely developed in MATLAB for initial validation. It is possible to record or choose a predefined movement. The range of motion (movement amplitude) can be changed by the therapist according to each patient in each session. The game goal is to take the ball to the basket by performing vertical movements. Early in the game, the ball, in the lower position, rises when an upward movement is performed. After reaching the basket, the ball position is reset, and the “hand” needs to return to pick it up. The force, measured by a load cell, is shown to the user in a gauge indicator, alerting the patient if its participation is unsatisfactory (orange or red) and scoring a lower value (1 point). If its participation is satisfactory, yellow, or green, 2 and 3 points are scored respectively. This game also applies an assist-as-needed control where the device assists the patient movement when its participation is not enough.

The Square Apple game, Figure 6B, was designed for the CAR in setup II. This game has also been fully developed in MATLAB. Square Apple follows the characteristics of a game with motion/virtual reality control, where a hand is moved on a plane to harvest apples, using as input the user’s movements up/down and left/right on the mobile platform. Each game session ends when the user picks up all the apples and puts them in the basket. The Grabbing Apple is an update from the previous game to the Unity platform for better interface and visual effects. In a variant of this game, Figure 6C, the patient focuses only on one apple at time what allow the application of assistance strategies.

The Paper Plane game, Figure 6D, was designed in Unity for the CAR in setup II. It is noteworthy that this game also works with the bimanual setup. In this game an airplane moves to dodge obstacles that fall from the top of the screen (Escape mode); or catches geometric shaped objects that constantly fall off the screen (Survival mode). Score is given as function of the playtime without losing and the game is over when an obstacle is hit (Escape) or an object is lost (Survival). Both game modes use horizontal movements (left/right) or bimanual rotation movements (CW/CCW) performed by the user on the handle/handlebar.

As shown in Section 2.5, the motor functions of stroke patients are often evaluated by circle drawing tasks. The Round Pizza game, Figure 6E, was developed in Unity and MATLAB and designed for setup II, but instead, it uses a circular trajectory. In this game a cutter is moved in order to cut the pizza dough in the most rounded way, using as input the patient’s movements (up/down and left/right combined) in the handle to perform a circular motion. Each session ends after completing a full circle, then the circularity and exercise time efficiency in each session can be compared. The pizza diameter is related with the movement amplitude/difficulty. Preliminary tests performed with the circular trajectory achieved an average error of 0,35 ± 0.27 cm which corresponds to 2,33 ± 1.83%, indicating good accuracy (Alves et al., 2019).

The Motorcycle game, Figure 6F, was designed for the CAR in setup III. Motorcycle follows the characteristics of a motion/virtual reality control game, in which a motorcycle must be driven down a track dodging obstacles and advancing on its route. When hitting an obstacle, the bike automatically returns to the starting position while the score is the traveled distance. The game input are the rotation movements (CW/CCW) performed by the user on the handlebars of the device, Figure 1C. The CW rotation of the real handlebar corresponds to the bike turning right, Figure 6F; CCW rotation turns left. To rotate the handlebar the user must indicate the intended movement by applying a force on a side greater than the other by a predefined value ΔF . To rotate clockwise, the force on the right side must be greater than the left by ΔF and vice versa to rotate counterclockwise. Movement can be facilitated by decreasing ΔF (assistive) or hampered by increasing ΔF (resistive).

**FIGURE 7:**
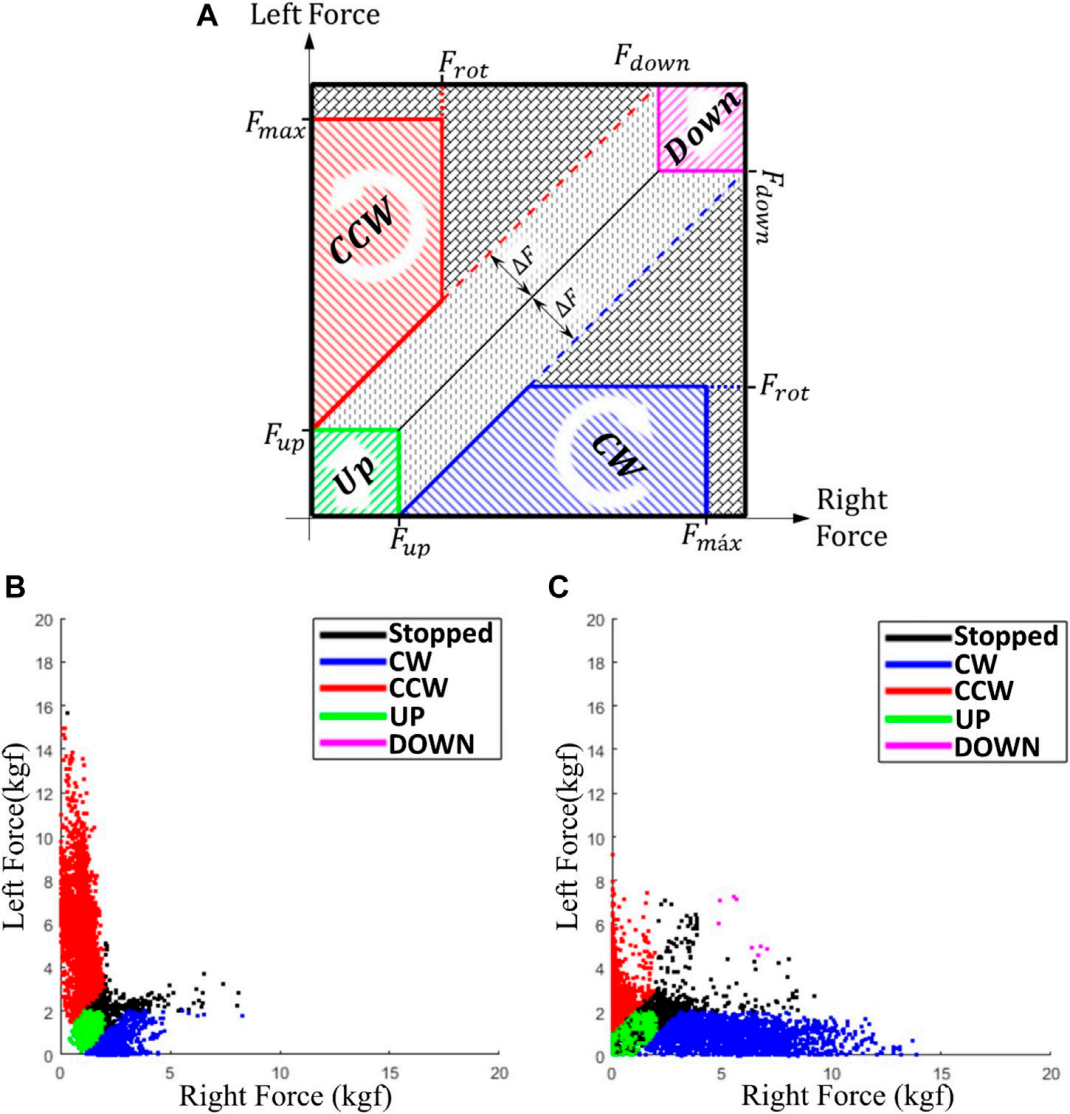
Force map graph for the bimanual serious games, **(A)** constraints and effects for rotation and translation movements, **(B)** activation distribution example of a right and **(C)** left hemiparetic subject.

The MineCart and Sakura Flowers games (Figures 6G,H, respectively) were also designed for use with the bimanual setup. In the MineCart game a wagon moves horizontally to pick up crystals that fall from the ceiling of a mine cave and uses the rotation movements (CW/CCW) as input. The rotation of the handlebar clockwise corresponds to the wagon moving to the right and the counterclockwise to the left. MineCart follows the same strategy for movement of the Motorcycle, but with 2 additional restrictions. First, to prevent the learned non-use (when patients no longer use the weaker limb and concentrate their efforts on the nonparetic side) the patients are required to always use the opposite arm to lift the bar until the force on this side is less than a predefined value, F_rot_. Another constraint considers when the patients could mistakenly put enough force on the wrong side and transfer it to the opposite side as they are coupled. Thus, a maximum force limit, F_max_, was assigned, acting as a warning and it is discouraging the patient from applying excessive forces on one side only. These constraints and their effects are shown on the force map graph in Figure 7A. Figure 7B shows an example of the force map activation distribution of a right hemiparetic subject during a session with this game; the Figure 7C shows an example of a left hemiparetic. Note that the subjects tend to use more the opposite side of their hemiparesis. MineCart score is proportional to the number of crystals caught. A hidden score for the patient counts the lost crystals and the balance of the last five crystals (caught/lost). This balance defines if assistance is required and if so, its level. According to its level, assistance acts by initially facilitating and then even performing the movement at the right direction by itself. In addition, undesirable movements are hampered/hindered. Additionally, this game uses a performance-based strategy for assistance. Resistive assistance (contrary to the movement direction) can be applied by choosing a negative assistance value and, consequently, making the handlebar rotation harder (heavier).

The MineCart game has three modes:

1) Constant: crystals at same speed; no changes when picking up more crystals (higher score);
2) Accelerated: crystal drop rate/speed increases with crystals caught (10% every 10 points);
3) Dynamic: resistive assistance applied (heavier movement) according to the score.

The movements of human limbs have great variability and, additionally, lack precision in stroke patients. Hence a tolerance, related to the desired position, is allowed. In the MineCart game, this tolerance is given by the wagon size related to the crystal. Therefore, the position does not need to be perfectly coincident with the desired position and any object within this range will be collected, furthermore avoiding patient’s frustration. Sakura Flowers Game, Figure 6H, follows the same game mechanics and features of Minecart. The basket/flowers are considerably smaller than wagon/crystals which makes this game becomes more difficult, requiring more accurate movements (smaller tolerance) than MineCart and acting as a next level for progression.

## 4 BiEval: Software for Evaluation and Follow-Up

Evaluation software can measure patient progress based on gains and quantifiable measures of time, speed, and smoothness of movement. To analyze the results of therapy sessions and to follow-up patient’s progress in a quantitative way, BiEval software was developed in Matlab App. During game execution, BiEval stores the data of each session, by date and time, of each patient. After, it provides the results and a history of the patient’s progress by using performance parameters.

The introduction of these parameters aims to condense the collected data, transforming them into information to quantitatively evaluate the effect of rehabilitation therapy, being also possible to compare them between sessions and/or patients/pathology. These parameters provide information regarding time, position (error), speed, movement smoothness, force and torque and allow for the quantitative analysis of movement characteristics. Additionally, the parameters are directly related to the clinical outcomes as the time, position error, speed and movement smoothness are related to the patient’s reaction, mobility and motor control and the force/torque to its strength.

### 4.1 Time Parameters

**FIGURE 8:**
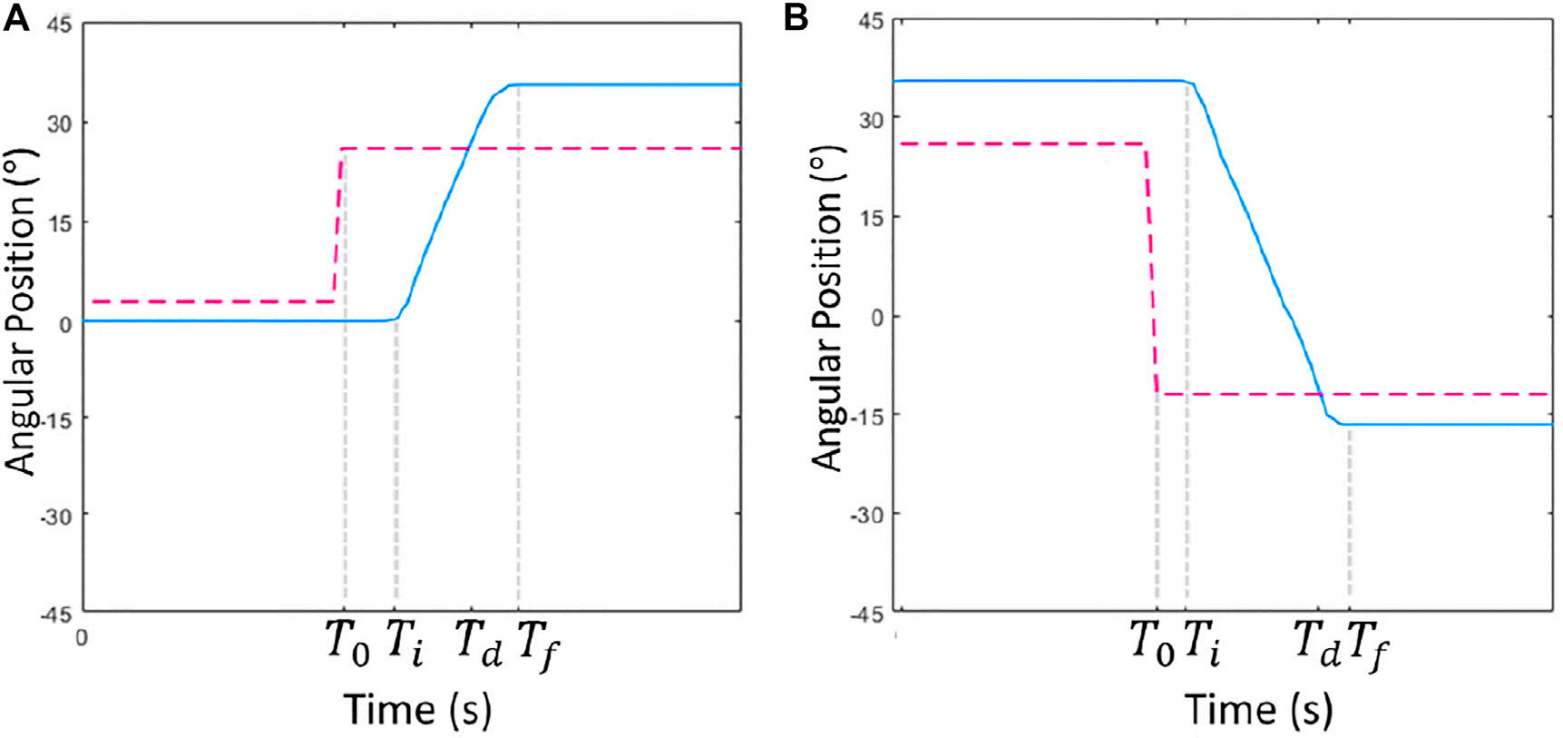
Points for calculating the time intervals, **(A)** counterclockwise, **(B)** clockwise movement. Blue and red are, respectively, the current and the desired position (*θ* and *θ*_d_).

The time parameters analyze the patient’s efficiency to initiate and complete the movement through the reaction time *T*_r_) and required time *T*_m_), respectively. *T*_r_ is defined as the interval elapsed since a new desired position *θ*_d_ and the patient’s onset of movement *T*_0_ and *T*_i_ respectively). In the MineCart game, *θ*_d_ is the position of the new crystal *T*_m_ is the time required to complete the movement since the beginning of movement *T*_i_ and the completion time of *T*_f_. These variables are shown in Figure 8A for a counterclockwise movement segment and Figure 8B for a clockwise movement segment. *T*_d_ is the instant the desired position is reached. The start time of movement *T*_i_ is defined as the moment when the patient performs a productive torque (positive if the effort is performed in the direction of the desired movement) and, in addition, the angular velocity exceeds a selected threshold. The final time *T*_f_ is defined as the moment after reaching the desired position, where the angular velocity is lower than a selected threshold, i.e., after reaching the position *θ*_d_, the patient does not change his position significantly.

The parameters are obtained through the average values, being necessary the previous segmentation of each repetition of movement performed by the patient. The patient’s improvement can be noticed visually comparing the segmentation of his moves at the beginning and end of the intervention. A validation is performed for the purpose of discarding inconsistent values (much higher or smaller). To avoid valid values being discarded (due to the wide range of human movement), the exclusion criterion is to be statistical outlier related to the peak values. This same validation will also be used for all parameters that will be shown in the sequence.

### 4.2 Speed Parameters

After the movement segmentation it is possible to calculate the average (*ω*_m_) and the peak angular velocity (*ω*_p_) of each repetition. These parameters demonstrate the patient speed in completing the exercise movement and is related to its mobility.

The average velocity *ω*_m_ corresponds to the average of the absolute value of angular velocity during the interval *T*_m_ of each movement (*T*_i_ to *T*_f_), i.e.:

$$\begin{array}{c} \omega_{m}=\frac{1}{T_{f}-T_{i}}\;\overset{T_{f}}{\underset{T_{i}}{\int }}\left|\omega \left(t\right)\right|dt \end{array}$$ (10)

where the angular velocity ω (t) was calculated as the first derivative of the angular position θ.

The peak velocity *ω*_p_ corresponds to the maximum absolute value reached by the angular velocity during the *T*_m_ interval of each movement:

$$\begin{array}{c} \omega_{p}=max\left(\left|\text{ω}\left(t\right)\right|\right)forT_{i}\leq t\leq T_{f} \end{array}$$ (11)

### 4.3 Force and Torque Parameters

**FIGURE 9:**
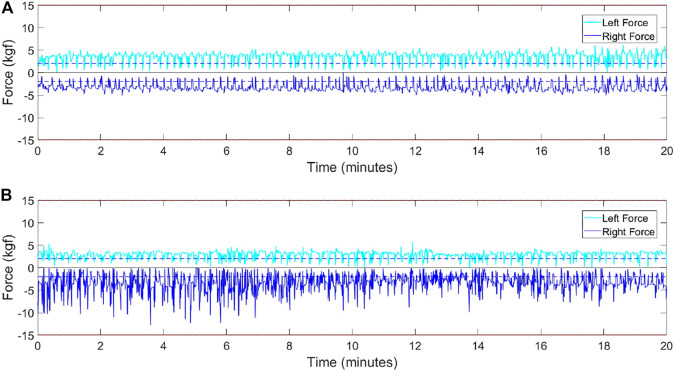
Force profiles: **(A)** healthy subject, **(B)** left hemiparetic subject.

The force and torque parameters are related to the patient strength, to its magnitude and direction, respectively. As seen, after a stroke the patient perform the “learned non-use” of the most impaired arm, concentrating his efforts on the nonparetic side. Compensation with the nonparetic side generally produces relatively larger peaks of forces on this side. Figure 9 shows the comparison of the strength profile of two participants: in (a) without paresis and (b) with left-sided hemiparesis. In Figure 9A, the force profile is symmetrical, more restrained and has few force peaks. In the Figure 9B unlike, the force profile is more irregular and there are more peaks on the right (subject’s nonparetic side). The peak force parameter *F*_p_) is therefore, defined as the average of the peak forces encountered with Matlab findpeaks functions:

$$\begin{array}{c} F_{p_{j}}=\frac{1}{n}\overset{n}{\underset{i=1}{\sum }}findpeaks\left[F_{j}\right] \end{array}$$ (12)

where *n* is the force peak number found on each side *j*. The peak force symmetry between the left and right sides (δF) is considered positive when the left peak forces is higher and negative otherwise (null if equal):

$$\begin{array}{c} \delta F=F_{p_{left}}-F_{p_{right}} \end{array}$$ (13)

Although like force parameters, the torque parameters consider the correct direction of effort for the desired position (productive torque ${\text{τ}}_{\text{p}}$ ) or penalties for wrong (counterproductive torque ${\text{τ}}_{\text{cp}}$ ). Total torque ( ${\text{τ}}_{\text{total}}$ ) is considered productive (positive) when the torque on both sides is productive. If the torque on one side is counterproductive (negative), the total torque is considered counterproductive. Finally, the torque symmetry between the sides ( δτ ) is defined as positive when the left torque is higher and negative otherwise.

### 4.4 Movement Smoothness Parameters

The normalized jerk *J*_n_, Eq. 14, is a parameter to quantify the smoothness of movement and corresponds to the ratio between the value of jerk (rate of change of acceleration) and the peak movement velocity *ω*_p_.

$$\begin{array}{c} J_{n}=\frac{1}{\omega_{p}}\left(\frac{1}{T_{f}-T_{i}}\right)\overset{T_{f}}{\underset{T_{i}}{\int }}J\left(t\right)dt \end{array}$$ (14)

Where the angular jerk, *J*(*t*), is defined as first derivative of the angular acceleration with respect to time:

$$\begin{array}{c} J\left(t\right)=\frac{d^{3}\text{Θ}}{dt^{3}} \end{array}$$ (15)

The number of velocity peaks *N*_p_ during the movement is also a smoothness parameter as patients with motor disabilities (spastic) tend to fractionate the movement constantly accelerating and decelerating (Roy et al., 2011; Ibarra, 2014), hence increasing *N*_p_ and $J_{n}$ . The number of peaks *N*_p_ is calculated using the Matlab findpeaks function:

$$\begin{array}{c} N_{p}=\frac{1}{T_{f}-T_{i}}\overset{T_{f}}{\underset{T_{i}}{\int }}\left(findpeaks\left[\omega \left(t\right)\right]\right)dt \end{array}$$ (16)

These parameters demonstrate the patient capability to produce a smooth-uninterruptible movement and are directly related to the patient motor control.

### 4.5 Error Parameters

The error parameter used was the quadratic mean of the position error, ${\bar{\text{Θ}}}_{\text{error}}^{\text{RMS}}$ . The quadratic mean of the error is defined as the square root of the mean position error ${\text{Θ}}_{\text{error}}$ squared during the *T*_m_ interval of each movement:

$$\begin{array}{c} {\bar{\text{Θ}}}_{error}^{RMS}={\left(\frac{1}{T_{f}-T_{i}}\overset{T_{f}}{\underset{T_{i}}{\int }}{\left(\text{Θ}\left(t\right)-{\text{Θ}}_{d}\left(t\right)\right)}^{2}dt\right)}^{\frac{1}{2}} \end{array}$$ (17)

This parameter indicates the patient accuracy to reach the desired position and keep on it for a while.

### 4.6 Performance Parameters Presentation

**FIGURE 10:**
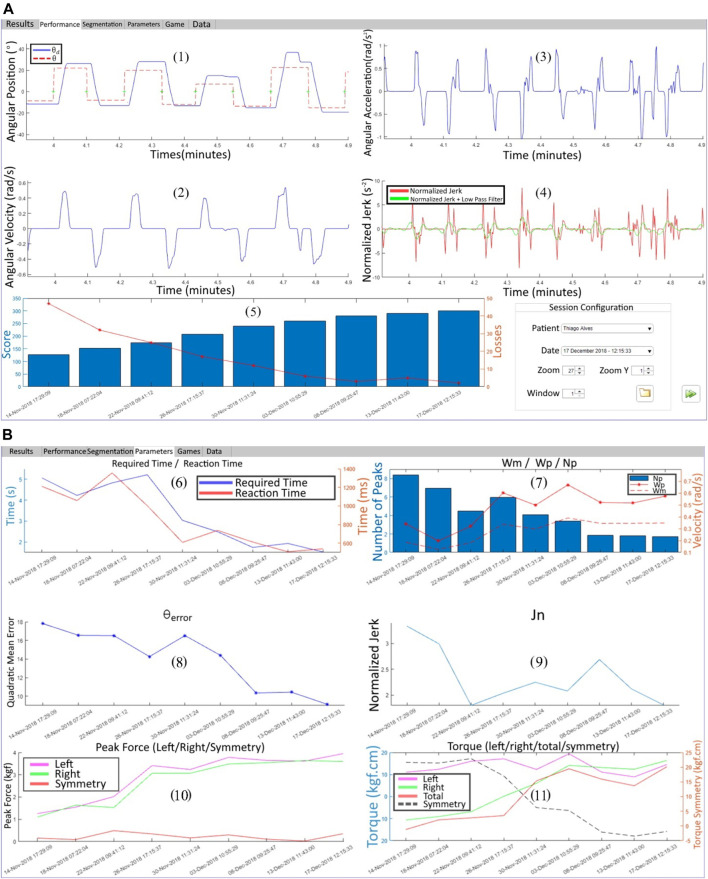
BiEval software: **(A)** first tab with (1) *θ* and *θ*_d_, (2) ω, (3) α, (4) *J*_n_ and (5) score evolution in each rehabilitation session, **(B)** performance parameters evolution.

In a tab of the BiEval software showed in Figure 10A, in addition to collected data, some mathematical and computational calculations are presented to obtain other information such as: 1) angular position (°) Θ and ${\text{Θ}}_{\text{d}}$ used for the calculation of *ω*, *α* and *J*_n_; 2) angular velocity *ω* in rad/s; 3) angular acceleration α in $\text{rad}/{\text{s}}^{2}$ ; 4) normalized angular jerk *J*_n_ in ${\text{s}}^{-2}$ and 5) evolution of the score (blue) and losses (red) in each rehabilitation game session.

Performance parameters are summarized in a tab of the software BiEval showing an example of patient’s progress through the many sessions.

The parameters are presented in Figure 10B and are, respectively: (6) *T*_m_ and *T*_r_; (7) *N*_p_, *ω*_m_ and *ω*_p_; (8) ${\bar{\text{Θ}}}_{error}^{RMS}$ ; (9) *J*_n_; (10) ${\text{F}}_{{\text{p}}_{\text{j}}}$ and ΔF and (11) ${\text{τ}}_{p_{\text{j}}}$ / ${\text{τ}}_{\text{cpj}}$ , ${\text{τ}}_{{\text{p}}_{\text{total}}}$ and δτ .

## 5 Clinical Tests

Patients who experience serious games in stroke rehabilitation have similar results in improving arm function and report greater engagement compared to conventional therapy. A RCT with 8 chronic stroke patients who received reach training using Freebal device and a serious game concluded that most patients improved the distance and the direction of reaching performance after training and they achieved higher Fugl-Meyer (FM) scores which is an index to assess the sensorimotor impairment in stroke patients (Prange, 2009). Another RCT in 7 rehabilitation centers with 70 patients with subacute stroke compared with ArmeoBoom^®^ training and a serious game with same intensity conventional therapy found similar gains in both groups regarding the FM score (Prange et al., 2013). In Kottink et al. (2014) a RCT with 20 chronic stroke patients compared the effect of reach training with a serious game with conventional training. Both groups significantly improved the function of arm (FM) and hand (Action Research Arm). All patients reported positive experience in an Intrinsic Motivation Inventory (IMI) performed but the game group achieved a better evaluation. In Prange et al. (2015), a multicenter RCT performed with 70 patients with subacute stroke and similar guidelines found that FM scores and the reach distance improved significantly in both groups, but the serious game’s group reported greater interest/fun than the conventional group.

This section presents a RCT performed to test the proposed bimanual robot and the evaluation software presented in this work. In this RCT the subjects were allocated into two groups: 15 healthy and 10 post-stroke subjects. First, the system operation was validated with the healthy group. Additionally, this group provides baseline values of movement to the post-stroke group comparison. The inclusion criteria were for the healthy group, men, or women over 18 years old without history of neurological or joint diseases; for the stroke group, men, or women over 18 years old with stroke diagnoses who attended the Physiotherapy’s Clinic of the Federal University of Uberlândia (UFU) for rehabilitation purposes. Exclusion criteria: subjects who felt discomfort while performing the movements or had severe visual impairment. The clinical tests were performed at the Physiotherapy’s Clinic/UFU, Minas Gerais, Brazil. The Research Ethics Committee of the UFU approved this study (CAAE N° 00914818.5.0000.5152) and written informed consent was obtained prior to any data collection.

### 5.1 Health Subjects Tests

The healthy subjects were 11 males and 4 females from 19 to 30 years old (mean 22.4 ± 2.8 years old). The healthy participants performed upper limb exercises using the MineCart game. Each participant achieved an average score of 200 points (approximately 200 repetitions).

After the tests with healthy participants using the MineCart game, the motivation was measured with an Intrinsic Motivation Inventory (IMI). IMI is a multi-dimensional questionnaire that provides qualitative information on the content and level of motivation a participant experiences during an intervention. It is scored on a 7-point Likert scale, ranging from “nothing true” to “very true” (“strongly disagree/agree”). A neutral IMI score is 4, and a higher score means a more positive motivation score (IMI, Intrinsic, 2021). The Interest/Satisfaction subscale is considered the self-reported measure of intrinsic motivation; Perceived Choice (patients only) and perceived competence are theorized as positive predictors of both self-report and behavioral measures of intrinsic motivation. Pressure/Tension is theorized as a negative predictor of intrinsic motivation.

A Game Engagement/Experience Questionnaire (GEQ) (IJsselsteijn, 2008) was applied to the group of healthy subjects to capture the experience of the player in terms of fun, frustration, and challenge. In the GEQ performed, the game is rated by the players (1–5) in the following categories: “Interface”, “Gameplay”, “Fun”, “Difficulty”, “Frustrating” and “Overall”.

### 5.2 Post-Stroke Patients Tests

**FIGURE 11:**
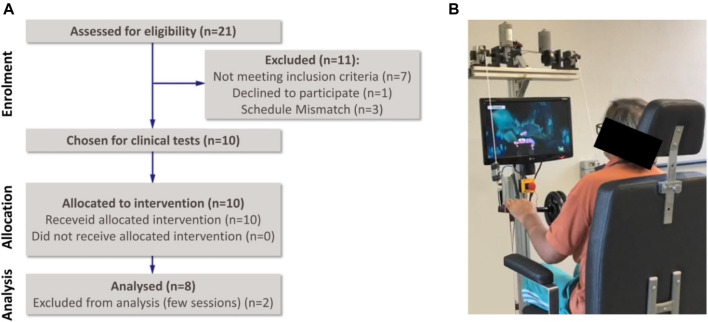
Clinical tests with post-stroke patients: **(A)** Consolidated Standards of Reporting Trials flow diagram, **(B)** system interfaced with a subject.

The post-stroke patients consisted of four males and six females from 39 to 70 years old (mean 58.4 ± 9.7 years), five was chronic stroke and five was acute stroke patients (6 ± 2.1 months after stroke). In addition, six patients had hemiparesis on the left side and four on the right side to some degree. Although all post-stroke patients were eligible, two patients reported tiredness and performed very few therapy sessions. Therefore, they were excluded from the post-intervention progress assessment and removed from the RCT that was filled with 8 remaining patients. The flow diagram of the clinical test with post-stroke patients is shown in Figure 11A.

The post-stroke patients participated in a 5-week bimanual robotic training program consisting of three 30-min sessions per week consisted of exercises using the BiCAR device and the MineCart game (constant mode), Figure 11B, combined with 50-min sessions of conventional rehabilitation therapy before robotic training. In each patient first robotic session, the handlebar height is adjusted for the movements related to the upper limbs. At the beginning of each session, the system adjusts itself, returning the handlebar to the initial position *θ* = 0° (horizontal). At any time during the game, the handlebar height can be readjusted for comfort; raising the bar with both hands or by applying force on both sides to adjust the handlebar position up or down, respectively (green/purple regions in Figure 7A).

The robotic training exercises consisted of rotation on the bimanual device, Figures 4D,E, mainly activating the upper limbs and requiring patients to use both arms together, forcing them to also use the weaker limb. For rotational movements it is necessary to decrease the length of one cable while increasing the length of the other. To do that the patients must indicate their intention of movement with both hands by applying a force in one side while lifting the other, according with Figure 7A. Additionally, the user is instructed by the therapist to not move the bar in the horizontal plane. This movement is not constrained however there is no issue if he/she pulls the systems outside of its predefined working plane.

During the exercise, the time required to perform each repetition, the force and the movement performed by the patient (among other calculated data shown in the parameters) were collected by the device for further evaluation. The effect of the therapy intervention was evaluated by comparing the patient performance parameters given by the BiEval software before and after the intervention, specifically at admission and discharge. An IMI was also applied to the group of post stroke patients.

## 6 Results

**FIGURE 12:**
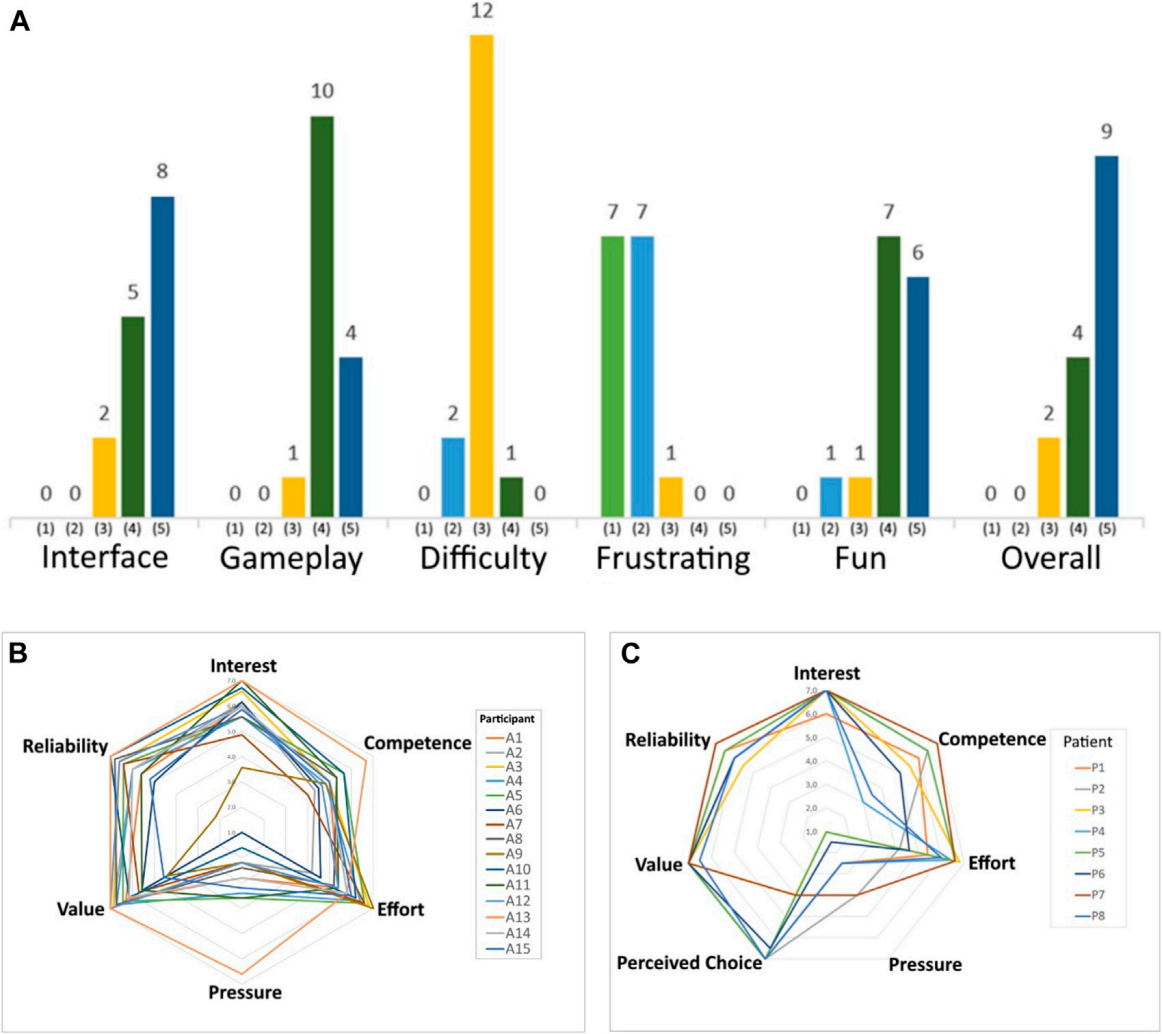
Questionnaires applied (indices in each category): **(A)** GEQ for healthy subjects, Intrinsic Motivation Inventory for **(B)** healthy subjects and **(C)** post-stroke patients.

The results of the GEQ performed is shown in Figure 12A. The average score for *Interface* was 4.4; for *Gameplay* and *Fun* was 4.2. The game *Difficulty* was considered average (2.9). The game was not considered *Frustrating* (1.6), and the *Overall* score was 4.5.

The indices in each IMI category performed with healthy subjects are presented in Figure 12B. Score close to 6 are observed in the categories Interest/Satisfaction (5.9 ± 0.9), Effort/Importance (5.8 ± 0.8), Value/Usefulness (6.1 ± 0.8) and Trust/Relationship (5.9 ± 1.2), indicating a high approval degree. While the Perceived Competence (5.1 ± 0.6) indicates that participants felt competent/skilled to a moderate level when performing the activity. Pressure/Tension (2.8 ± 1.3) is a negative predictor and indicates that they were not nervous, tense, or anxious during the exercises.

In the same way, the indices in each IMI category performed with post-stroke patients are presented in Figure 12C. Score close to 6 or 7 are observed in the categories Interest/Satisfaction (6.9 ± 0.4), Effort/Importance (5.8 ± 1.0), Value/Usefulness (6.9 ± 0.2) and Trust/Relationship (6.3 ± 0.5), indicating a high approval degree. The Perceived Competence (5.4 ± 1.5) indicates that patients felt competent/skilled to a moderate level when performing the activity. The perceived choice (6.2 ± 1.4) demonstrates that patients understood the robotic training as their own choice. Pressure/Tension (2.4 ± 1.2) indicates that they also were not nervous, tense, or anxious during the exercises.

After performing the exercises, the average reaction and movement time were found for healthy subjects to use as a reference for post-stroke participants and to discover how different these values are between groups. For this purpose, the software BiEval was used, developed for analyzing the results and monitoring the user’s progress.

**FIGURE 13:**
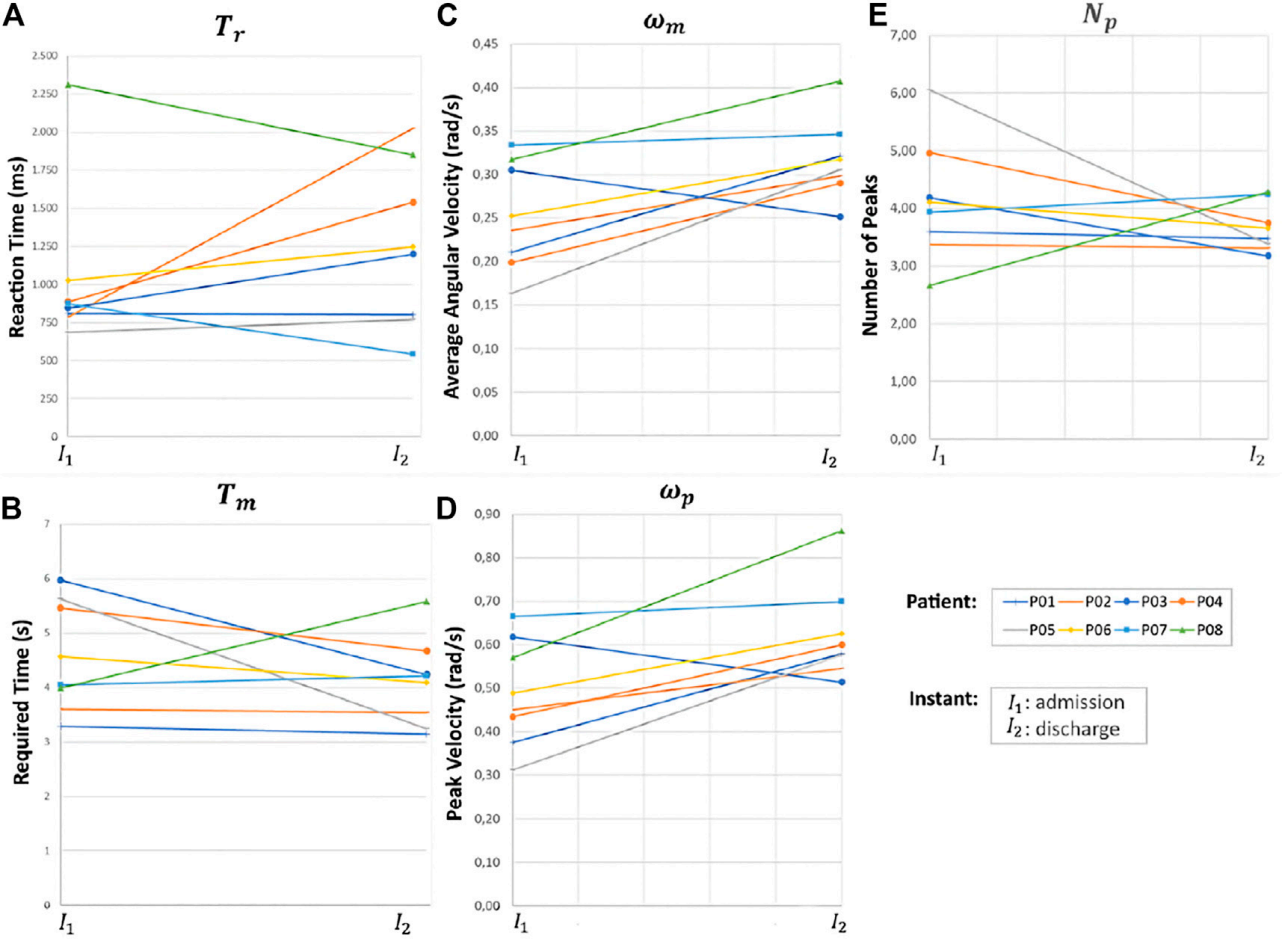
Patients performance parameters between the admission-discharge of the RCT, **(A)** *T*_r_, **(B)** *T*_m_, **(C)** *ω*_m_, **(D)** *ω*_p_, and **(E)** *N*_p_.

The average reaction time (*T*_r_) for the healthy participants was 728 ± 181 ms and the average time to complete the movement *T*_m_) was 2.5 ± 0.4 s. Regarding the effects of therapy intervention in post-stroke patients, from admission to discharge, the reaction time parameter, *T*_r_, increased in five patients and decreased in two (one patient had no changes). The average reaction time parameter increased from 1029 ± 569 ms to 1247 ± 574 ms, Figure 13A. Conversely, the time to complete the movement *T*_m_ (Figure 13B) decreased in six patients and increased in one. The average *T*_m_ decreased from 4.6 ± 1.1 s to 4.1 ± 0.9 s.

The angular velocity parameters, *ω*_m_ and *ω*_p_, increased in seven patients and decreased in one, Figures 13C,D, respectively. The average *ω*_m_ values before and after the intervention were 0.25 ± 0.07 rad/s and 0.32 ± 0.05 rad/s, respectively. The average *ω*_p_ before and after were 0.49 ± 0.13 rad/s and 0.62 ± 0.12 rad/s, respectively.

Regarding the movement smoothness parameters, the number of velocity peaks, *N*_p_, decreased in six patients (Figure 13E); two patients were having difficulty making the movement in the desired direction consequently, increasing the number of necessary movements. The average *N*_p_ before and after the intervention was 4.1 ± 1.1 peaks and 3.7 ± 0.4 peaks, respectively. Game scores increased post-intervention for all but one patient (155 ± 65 points to 199 ± 88 points), and the number of losses decreased for all patients (40 ± 12 losses to 17 ± 15 losses). These values indicate better mobility and control motor as healthier subjects have higher scores and less losses.

Both the right and left forces increased from 2.82 ± 1.14 kgf to 3.74 ± 0.59 kgf and 2.73 ± 1.06 kgf to 3.48 ± 0.69 kgf, respectively. The force symmetry parameter (δF) decreased in all patients, indicating that despite increasing the force applied on both sides, they learned how to use both arms more evenly; δF ranged from 0.94 ± 0.63 kgf to 0.7 ± 0.63 kgf. The other parameters did not improve significantly.

In the torque parameter, it was observed that patients with left hemiparesis tended to use more the right arm to compensate and vice versa, as predicted in Section 4.3. This is because the stroke patient concentrates his efforts on the non-paretic side during exercises producing relatively greater peak forces on this side, the “learned non-use” of the impaired limb. Thus, for the left hemiparetic patients, the average left torque was 7.55 ± 5.69 kgf.cm and the right was 12.59 ± 6.87 kgf.cm productive, for right hemiparesis, the right torque was 6.56 ± 4.87 kgf.cm and the left was 17.86 ± 5.48 kgf.cm productive.

**FIGURE 14:**
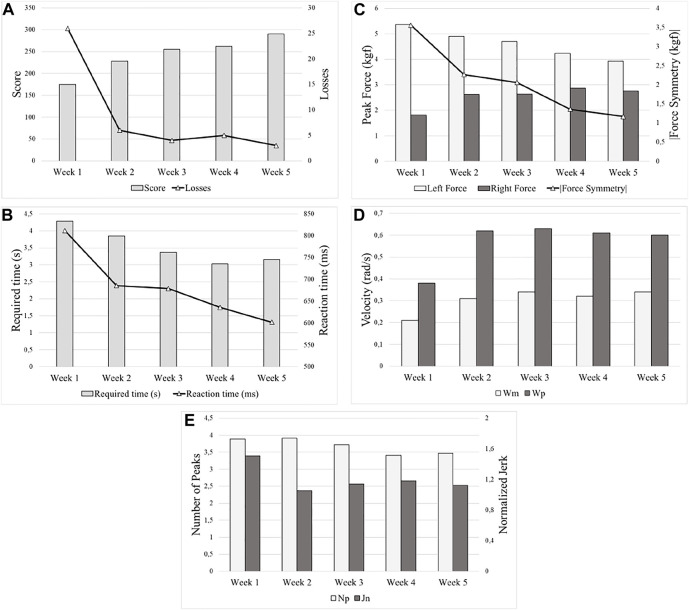
Improvement over time (week by week) of a post-stroke subject under the bimanual approach, **(A)** score and losses, **(B)** required and reaction time *T*_m_ and *T*_r_, **(C)** forces ${\text{F}}_{{\text{p}}_{\text{left}}}$ , ${\text{F}}_{{\text{p}}_{\text{right}}}$ , and δF, **(D)** velocity *ω*_m_ and *ω*_p_, and **(E)** movement smoothness parameters *N*_p_ and *J*_n_.

The data provided by the evaluation software are suitable to show the changes in the patient’s parameters over time using the bimanual approach. Figure 14 shows the improvement week by week in a post-stroke subject under bimanual intervention in: (a) score and losses, (b) required and reaction time (*T*_m_ and *T*_r_), (c) forces ${\text{F}}_{{\text{p}}_{\text{left}}}$ , ${\text{F}}_{{\text{p}}_{\text{right}}}$ , and δF, (d) velocity *ω*_m_ and *ω*_p_, and (e) movement smoothness *N*_p_ and *J*_n_.

## 7 Discussion and Conclusion

The use of robots in stroke rehabilitation allows a greater intensity of therapies, generating improvements in motor and functional performance. Serious games can be used together with these robots to enhance the progress tracking and provides an objective measures of therapy outcomes. Literature shows that training with serious games is as effective as conventional therapy in improving the arm’s function when applied at the same intensity. Besides that, patients using serious games reported positive experiences such as greater interest and fun. Cable-driven robots can be a good solution for stroke rehabilitation as these devices meet the requirements for safety, comfort, and portability, besides having a low cost. Additionally, the cable driven robots have as advantage not intimidating the patient. However, most cable driven robots are unilateral devices only and with therapies focusing on the most affected limb, neglecting bimanual activities which are predominantly related to activities of daily living. In this way, were presented the developments of the BiCAR, a bimanual cable-driven robot to be applied in stroke rehabilitation, a serious game approach to motivate the patients, and the BiEval, a software to quantitatively evaluate rehabilitation therapy effects.

To test the proposed robotic device BiCAR with a serious game and the follow-up approach with the BiEval software, a Randomized Controlled Trial was performed with two groups: 15 healthy and 10 post-stroke subjects. The post-stroke patients performed 3 sessions per week over a 5-week interval consisted of 30 min of bimanual robotic therapy with a serious game after 50 min of conventional rehabilitation therapy. Two patients were excluded from the evaluation due to reported tiredness.

The participant’s motivation was measured with an Intrinsic Motivation Inventory. The score values indicated a high approval degree, that the patients felt competent/skilled when performing the activity and were not nervous, tense, or anxious during the exercises.

The improvement of the parameters given by the BiEval software is directly related to the patient’s improvement in reaction time, precision, motor control, mobility, and strength. It was observed that there are parameters that can show differences between people’s mobility. The reaction time *T*_r_ and the time to complete the movement *T*_m_ were both greater in the post-stroke group than those presented by healthy participants (41% and 84% respectively related to both groups' first session), as expected due to the movement loss in stroke patients. Regarding the increase of the reaction time parameter in post stroke patients, we believe that when a patient realizes that he/she does not need to react so quickly, he or she will decrease attention on the activity and consequently increase the reaction time but decreasing in the time to complete the movement, *T*_m_, indicates better mobility to perform the movement in a shorter time at discharge. The decreased number of velocity peaks at discharge indicates a more continuous/smoother movement on those patients after the training. Finally, the better game score/losses after the training also indicates better mobility and control motor as healthier subjects have higher scores and less losses.

In general, the software can reveal movement differences after several rehabilitation sessions and measure how the movement was more continuous/smoother on those patients after the training. Game scores values can also indicate better mobility and control motor as healthier subjects has higher scores and less losses. The hemiparesis can be measured based on the distribution of force/torque on both sides and on the “learned non-use”. It also shows when there is a noticeable improvement in the use of both arms more evenly by the patients. The results showed that seven patients improved at least 10 out of 15 performance parameters given by the BiEval software after robotic therapy (one improved by only eight), and on average patients improved in 12 parameters. However, changes between the admission-discharge interval were significantly different (better) only for seven parameters: *ω*_m_, *ω*_p_, $F_{p_{right}}$ , $F_{p_{left}}$ , δF, scores, and losses. For the other parameters, there was no significant improvement. For all statistical analyses a significance of 0.05 was used.

Among the several serious games options presented the bimanual ones offer a totally new way to improve the patient’s ability focused on the ADLs. Additionally, all the games were developed for the cable driven device, which makes the proposed device unique in this field. Thus, using the robotic rehabilitation and the serious games we expect that patients can perform training at home, or even in the rehabilitation clinic, without relying on the therapist’s availability, possibly allowing an increased training dose in relation to supervised treatment and, consequently, a greater improvement in dexterity. The BiEval Software can be a tool to be used together to the physiotherapy to measure patient’s performance and progress in a more quantifiable way while helping post-stroke patients in the ability to use both hands in the activities of daily living. In this way, BiCAR/BiEval with serious games approach proves to be a feasible solution to increase the presence of robots in rehabilitation clinics around the world and to motivate the patients during the exercises making therapy more attractive and pleasurable, and softening the notion of effort.

Future research it is intended to increase the number of patients while including a clinical scale to compare the bimanual approach with other interventions. For future RCT implementations, it is intended to gradually reduce the time available for reaction (increase “objects’ gravity”), or to award faster reactions to incentive this behavior (bonuses). It is also planned to use more games while still allowing the patient to choose the games they want to play. Additionally, games will be developed by considering the neuroplasticity and cognitive/memory concepts.

## Data Availability

The original contributions presented in the study are included in the article/supplementary material, further inquiries can be directed to the corresponding author.

## References

[B1] Aguiar P. M. Burdet E. Caurin G. A. P. (2017). “Instrumented Module for Investigation of Contact Forces for Use in Rehabilitation and Assessment of Bimanual Functionalities,” in 24th ABCM International Congress of Mechanical Engineering.

[B2] Aguilar-Lazcano C. A. Rechy-Ramirez E. J. Hu H. Rios-Figueroa H. V. Marin-Hernandez A. (2019). Interaction Modalities Used on Serious Games for Upper Limb Rehabilitation: A Systematic Review. Games Health J. 8 (5), 313–325. 10.1089/g4h.2018.0129 31287734

[B3] Alves T. Gonçalves R. S. Carbone G. Ceccarelli M. (2019). “Cable-Driven Robots for Circular Trajectory Exercises in Rehabilitation,” in 25th ABCM International Congress of Mechanical Engineering. Uberlândia, MG, Brazil.

[B4] Appel V. C. R. (2014). “Classifying Emotions in Rehabilitation Robotics Based on Facial Skin Temperature,” in 5th IEEE RAS International Conference on Biomedical Robotics and Biomechatronics, 276.

[B5] Auriat A. M. Neva J. L. Peters S. (2015). A Review of Transcranial Magnetic Stimulation and Multimodal Neuroimaging to Characterize post-stroke Neuroplasticity. Front. Neurol. 6, 1–20. 10.3389/fneur.2015.00226 26579069 PMC4625082

[B6] Ballesteros S. Mayas J. Prieto A. Toril P. Pita C. Laura P. d. L. (2015). A Randomized Controlled Trial of Brain Training with Non-action Video Games in Older Adults: Results of the 3-month Follow-Up. Front. Aging Neurosci. 7, 45. 10.3389/fnagi.2015.00045 25926790 PMC4396447

[B7] Boschetti G. Carbone G. Passarini C. (2019). Cable Failure Operation Strategy for a Rehabilitation Cable-Driven Robot. Robotics. 8, 17. 10.3390/robotics8010017

[B8] Brodie S. M. Meehan S. Borich M. R. Boyd L. A. (2014). 5 Hz Repetitive Transcranial Magnetic Stimulation over the Ipsilesional Sensory Cortex Enhances Motor Learning after Stroke. Front. Hum. Neurosci. 8, 143. 10.3389/fnhum.2014.00143 24711790 PMC3968757

[B9] Burke J. W. McNeill M. D. J. Charles D. K. Morrow P. J. Crosbie J. H. McDonough S. M. (2009). Optimising Engagement for Stroke Rehabilitation Using Serious Games. Vis. Comput. 25, 1085–1099. 10.1007/s00371-009-0387-4

[B10] Cafolla D. Russo M. E. Carbone G. (2019). CUBE, a Cable-driven Device for Limb Rehabilitation. J. Bionic Eng. 16 (No. 2), 492. 10.1007/s42235-019-0040-5

[B11] Carbone G. Ceccarelli M. (2016). Sistema a Cavi Per Assistenza Motoria. (Cable Driven System for Motion Assistance). patent Appl. n.102016000038975.

[B12] Carbone G. Gherman B. Ulinici I. Vaida C. Pisla D. (2018). “Design Issues for an Inherently Safe Robotic Rehabilitation Device,” in *Advances in Service and Industrial Robotics. RAAD 2017. Mechanisms and Machine Science*. Editors Ferraresi, C., Quaglia, G. (Cham: Springer), Vol. 49. 10.1007/978-3-319-61276-8_110

[B13] Cardoso L. R. L. Matelleto M. N. Aguiar P. M. (2017). ““Upper Limb Rehabilitation Through Bicycle Controlling,”,” in 24th ABCM International Congress of Mechanical Engineering.

[B14] Caurin G. A. Siqueira A. A. Andrade K. O. Joaquim R. C. Krebs H. I. (2011). Adaptive Strategy for Multi-User Robotic Rehabilitation Games. Annu. Int. Conf. IEEE Eng. Med. Biol. Soc. 2011, 1395–1398. 10.1109/IEMBS.2011.6090328 22254578

[B15] Ceccarelli M. Riabtsev M. Fort A. Russo M. Laribi M. A. Urizar M. (2021). Design and Experimental Characterization of L-CADEL V2, an Assistive Device for Elbow Motion. Sensors. 21, 5149. 10.3390/s21155149 34372386 PMC8347154

[B16] Ceccarelli M. Romdhane L. (2010). Design Issues for Human-Machine Platform Interface in Cable-Based Parallel Manipulators for Physiotherapy Applications. J. Zhejiang Univ. Sci. A 11, 231–239. 10.1631/jzus.A1000027

[B17] Dedoncker J. Brunoni A. R. Baeken C. Vanderhasselt M.-A. (2016). The Effect of the Interval-Between-Sessions on Prefrontal Transcranial Direct Current Stimulation (tDCS) on Cognitive Outcomes: a Systematic Review and Meta-Analysis. J. Neural Transm. 123, 1159–1172. 10.1007/s00702-016-1558-x 27145765

[B18] Dipietro L. Krebs H. I. Fasoli S. E. Volpe B. T. Stein J. Bever C. (2007). Changing Motor Synergies in Chronic Stroke. J. Neurophysiol. 98, 757–768. 10.1152/jn.01295.2006 17553941

[B19] Eggers O. (1984). Occupational Therapy in the Treatment of Adult Hemi-Plegia. London, U.K: Heinemann.

[B20] Ferreira B. Menezes P. (2020). An Adaptive Virtual Reality-Based Serious Game for Therapeutic Rehabilitation. Int. J. Online Biomed. Eng. 16 (4), 63–71. 10.3991/ijoe.v16i04.11923

[B21] Gonçalves R. S. Alves T. Carbone G. Ceccarelli M. (2020). Cable-Driven Robots in Physical Rehabilitation. Adv. Comput. Intelligence Robotics, 52–96. 10.4018/978-1-7998-1382-8.ch003

[B22] Hatem S. M. Saussez G. Della Faille M. (2016). Rehabilitation of Motor Function after Stroke: A Multiple Systematic Review Focused on Techniques to Stimulate Upper Extremity Recovery. Front. Hum. Neurosci. 10, 1–22. 10.3389/fnhum.2016.00442 27679565 PMC5020059

[B23] Hocoma (2019). Armeo® Therapy Concept. Available at: https://products.iisartonline.org/products/24/marketing/BRO_Armeo_Therapy_Concept_130225_en.pdf (Accessed January 15, 2019).

[B24] Hussain A. Budhota A. Contu A. (2017). ““Quantitative Assessment of Motor Functions post-stroke: Responsiveness of Upper-Extremity Robotic Measures and its Task Dependence,” in IEEE International Conference on Rehabilitation Robotics, 1037–1042. 10.1109/ICORR.2017.8009386 28813958

[B25] Ibarra J. C. P. (2014). “Adaptive Impedance Control Applied to Robotic Ankle Rehabilitation,” Mather Thesis. São Paulo, Brazil: São Paulo University.

[B26] 80601-2-78:2019 IEC, IEC (2019). Medical Electrical Equipment. Geneva, Switzerland: IEC.

[B27] IJsselsteijn W. A. (2008). “Measuring the Experience of Digital Game Enjoyment,” in 6th International Conference on Methods and Techniques in Behavioral Research, Noldus Information Tecnology Wageningen (Netherlands: Maastricht), 88–89.

[B28] IMI, Intrinsic (2021). Motivation Inventory (IMI). Available at: http://selfdeterminationtheory.org/intrinsic-motivation-inventory (Accessed February 13, 2022).

[B29] InMotion (2021). Helps Traumatic Brain Injury Patient’s - Bionik Labs. Available at: https://www.bioniklabs.com/products/inmotion-arm (Accessed April 22, 2021).

[B30] Johnson M. J. Van der Loos H. F. M. Burgar C. G. Shor P. Leifer L. J. (2005). Experimental Results Using Force-Feedback Cueing in Robot-Assisted Stroke Therapy. IEEE Trans. Neural Syst. Rehabil. Eng. 13, 335–348. 10.1109/tnsre.2005.850428 16200757

[B31] Kottink A. I. Prange G. B. Krabben T. Rietman J. S. Buurke J. H. (2014). Gaming and Conventional Exercises for Improvement of Arm Function After Stroke: A Randomized Controlled Pilot Study. Games Health J. 3, 184–191. 10.1089/g4h.2014.0026 26196178

[B32] Krabben T. Prange G. B. Molier B. I. Stienen A. H. A. Jannink M. J. A. Buurke J. H. (2012). Influence of Gravity Compensation Training on Synergistic Movement Patterns of the Upper Extremity after Stroke, a Pilot Study. J. NeuroEngineering Rehabil., 9–44. 10.1186/1743-0003-9-44 PMC3443435 22824488

[B33] Krebs H. I. Bruce V. Hogan N. (2009). A Working Model of Stroke Recovery from Rehabilitation Robotics Practitioners. J. NeuroEngineering Rehabil. 6. 10.1186/1743-0003-6-6 PMC2649944 19243615

[B34] Laribi M. A. Carbone G. Zeghloul S. (2019). On the Optimal Design of Cable Driven Parallel Robot with a Prescribed Workspace for Upper Limb Rehabilitation Tasks. J. Bionic Eng. 16, 503–513. 10.1007/s42235-019-0041-4

[B35] Lüdemann-Podubecká J. Bösl K. Theilig S. Wiederer R. Nowak D. A. (2015). The Effectiveness of 1Hz rTMS over the Primary Motor Area of the Unaffected Hemisphere to Improve Hand Function after Stroke Depends on Hemispheric Dominance. Brain Stimulation. 8 (4), 823–830. 10.1016/j.brs.2015.02.004 25828427

[B36] Malabet H. G. Robles R. A. Reed K. B. (2010). “Symmetric Motions for Bimanual Rehabilitation,” in IEEE/RSJ International Conference on Intelligent Robots and Systems (Taipei: Taiwan).

[B37] Mao Y. (2015). “Human Movement Training with a Cable Driven ARm EXoskeleton (CAREX),” in IEEE Transactions on Neural Systems and Rehabilitation Engineering. 10.1109/tnsre.2014.2329018 24919202

[B39] Mehrholz J. Hädrich A. Platz T. Kugler J. Pohl M. (2012). Electromechanical and Robot-Assisted Arm Training for Improving Generic Activities of Daily Living, Arm Function, and Arm Muscle Strength after Stroke. Cochrane Database Syst. Rev. 6, CD006876. 10.1002/14651858.CD006876.pub3 22696362

[B40] Mishra J. Anguera J. A. Gazzaley A. (2016). Video Games for Neuro-Cognitive Optimization. Neuron. 90, 214–218. 10.1016/j.neuron.2016.04.010 27100194

[B41] Moretti C. B. Andrade K. O. Caurin G. A. P. (2014). Physiotherapy Support Web-Based System for Rehabilitation Robotics: An Initial Architecture. ABCM Symp. Ser. Mechatronics. 6.

[B42] Mubin O. Alnajjar F. Al Mahmud A. Jishtu N. Alsinglawi B. (2020). Exploring Serious Games for Stroke Rehabilitation: a Scoping Review. Disabil. Rehabil. Assistive Technology., 1–7. 10.1080/17483107.2020.1768309 32508187

[B43] Nijenhuis S. M. Prange G. B. Amirabdollahian F. S. P. (2015). Feasibility Study into Self-Administered Training at home Using an Arm and Hand Device with Motivational Gaming Environment in Chronic Stroke. J. NeuroEngineering Rehabil., 12–89. 10.1186/s12984-015-0080-y PMC4599772 26452749

[B44] Prange G. B. (2009). An Explorative Study into Changes in Reach Performance After Gravity Compensation Training in Chronic Stroke Patients. Japan: IEEE 11th ICORR. 10.1109/ICORR.2011.5975402 22275605

[B45] Prange G. B. Kottink A. I. R. Buurke J. H. Rietman J. S. (2013). “Application of Arm Support Training in Sub-acute Stroke Rehabilitation: First Results on Effectiveness and User Experience,” in IEEE International Conference on Rehabilitation Robotics (Seattle: Washington USA), 24–26. 10.1109/ICORR.2013.6650470 24187287

[B46] Prange G. B. Kottink A. I. R. Buurke J. H. Eckhardt M. M. E. M. van Keulen-Rouweler B. J. Ribbers G. M. (2015). The Effect of Arm Support Combined With Rehabilitation Games on Upper-Extremity Function in Subacute Stroke. Neurorehabil. Neural Repair. 29, 174–182. 10.1177/1545968314535985 24878589

[B47] Proença J. P. Quaresma C. Vieira P. (2018). Serious Games for Upper Limb Rehabilitation: a Systematic Review. Disabil. Rehabil. Assistive Technology. 13, 95–100. 10.1080/17483107.2017.1290702 28359181

[B48] Rosati G. Gallina P. Masiero S. (2005). ““Design of a New 5 d.o.F. Wire-Based Robot for Rehabilitation,” in Proceedings - 9th International Conference on Rehabilitation Robotics, 430–433.

[B49] Rosati G. Masiero S. Rossi A. (2017). On the Use of Cable-Driven Robots in Early Inpatient Stroke Rehabilitation,” in Advances in Italian Mechanism Science. Mechanisms and Machine Science, vol. 47. Editors Boschetti G. Gasparetto A. (Cham: Springer). 10.1007/978-3-319-48375-7_59

[B50] Roy A. Forrester L. W. Macko R. F. (2011). Short-term Ankle Motor Performance with Ankle Robotics Training in Chronic Hemiparetic Stroke. J. Rehabil. Res. Development. 48, 417–429. 10.1682/jrrd.2010.04.0078 21674391

[B51] Russo M. Ceccarelli M. (2020). Analysis of a Wearable Robotic System for Ankle Rehabilitation. Machines. 8, 48. 10.3390/machines8030048

[B70] Stefano M. Patrizia P. Mario A. Ferlini G. Rizzello R. Rosati G. (2014). Robotic Upper Limb Rehabilitation After Acute Stroke by NeReBot: Evaluation of Treatment Costs. Biomed. Res. Int. 2014, 265634. 10.1155/2014/265634 24967345 PMC4017845

[B52] Trlep M. Mihelj M. Puh U. Munih M. (2011). Rehabilitation Robot with Patient-Cooperative Control for Bimanual Training of Hemiparetic Subjects. Adv. Robotics. 25, 1949–1968. 10.1163/016918611x588853

[B53] Tyromotion, Diego (2021). Available at: https://tyromotion.com/en/produkte/diego/ (Accessed April 22, 2021).

[B54] Valentin L. S. S. (2017). Can Digital Games Be a Way of Improving the Neuroplasticity in Stroke Damage? Can the Adult Brain Grow New Cells or Rewire Itself in Response to a New Experience? Open J. Med. Psychol. 06, 153–165. 10.4236/ojmp.2017.62013

[B55] World Health Organization (2021). Available at: https://www.who.int/ (Accessed February 03, 2021).

[B56] Zhang M. Claire D. T. Xie S. (2013). Effectiveness of Robot-Assisted Therapy on Ankle Rehabilitation A Systematic Review J. NeuroEng. Rehab. BioMed Central 10, n.1. 10.1186/1743-0003-10-30 PMC3636117 23517734

